# Pan-genome association study of *Mycobacterium tuberculosis* lineage-4 revealed specific genes related to the high and low prevalence of the disease in patients from the North-Eastern area of Medellín, Colombia

**DOI:** 10.3389/fmicb.2022.1076797

**Published:** 2023-01-04

**Authors:** Uriel Hurtado-Páez, Nataly Álvarez Zuluaga, Rafael Eduardo Arango Isaza, Bruno Contreras-Moreira, François Rouzaud, Jaime Robledo

**Affiliations:** ^1^Corporación para Investigaciones Biológicas (CIB), Medellín, Colombia; ^2^Facultad de Ciencias, Universidad Nacional de Colombia (UNAL), Medellín, Colombia; ^3^Estación Experimental de Aula Dei–Consejo Superior de Investigaciones Científicas (EEAD-CSIC), Zaragoza, Spain; ^4^Fundación ARAID, Zaragoza, Spain; ^5^Ministry of Agriculture, Occitania, France; ^6^Escuela de Ciencias de la Salud, Universidad Pontificia Bolivariana (UPB), Medellín, Colombia

**Keywords:** *M. tuberculosis*, sublineage, transmission, prevalence, pan-GWAS, pan-genome, variant

## Abstract

*Mycobacterium tuberculosis* (*Mtb*) lineage 4 is responsible for the highest burden of tuberculosis (TB) worldwide. This lineage has been the most prevalent lineage in Colombia, especially in the North-Eastern (NE) area of Medellin, where it has been shown to have a high prevalence of LAM9 SIT42 and Haarlem1 SIT62 sublineages. There is evidence that regardless of environmental factors and host genetics, differences among sublineages of *Mtb* strains play an important role in the course of infection and disease. Nevertheless, the genetic basis of the success of a sublineage in a specific geographic area remains uncertain. We used a pan-genome-wide association study (pan-GWAS) of 47 *Mtb* strains isolated from NE Medellin between 2005 and 2008 to identify the genes responsible for the phenotypic differences among high and low prevalence sublineages. Our results allowed the identification of 12 variants in 11 genes, of which 4 genes showed the strongest association to low prevalence (*mmpL12*, *PPE29*, *Rv1419*, and *Rv1762c*). The first three have been described as necessary for invasion and intracellular survival. Polymorphisms identified in low prevalence isolates may suggest related to a fitness cost of *Mtb*, which might reflect a decrease in their capacity to be transmitted or to cause an active infection. These results contribute to understanding the success of some sublineages of lineage-4 in a specific geographical area.

## Introduction

Tuberculosis (TB) is a major public health problem worldwide and the most prevalent infectious-contagious disease in the history of humankind (Galagan, 2014; Couvin and Rastogi, 2015; World Health Organization [WHO], 2018). According to the World Health Organization’s (WHO’s) “Global TB report 2022” the TB is one of the leading causes of death from a single infectious agent (above HIV/AIDS). Globally, in 2021 there were an estimated 10.6 million new cases with active disease and about 1.4 million deaths (World Health Organization [WHO], 2022). The disease is caused by members of the *Mycobacterium tuberculosis* complex (MTBC), which includes five human-adapted lineages representing *Mtb* sensu stricto, L1 (The Philippines and Indian Ocean), L2 (East Asia), L3 (India and East Africa), L4 (Europe and Americas), and L7 (Ethiopia), and two other human-adapted lineages defined to as *M. africanum* L5 (West African 1), L6 (West African 2), and more than eight animal-adapted lineages globally distributed, which have evolved from common ancestors from different geographic areas (Ngabonziza et al., 2020). There are studies in MTBC that have associated genotypes with different clinical presentations of the disease, the geographic distribution, and their prevalence (David et al., 2012; Merker et al., 2015). Indeed, it has been shown that modern lineages (L2 and L4) are more successful in their spread capacity, being more prevalent than ancient lineages (L1, L3, L5, and L6), likely due to greater virulence and shorter latency periods (Gonzalo-Asensio et al., 2018; Ngabonziza et al., 2020).

However, although modern *Mtb* lineages are more virulent and spread faster, their sublineages do not always behave similarly. It partially depends on environmental factors such as antibiotic resistance, host demography, and genetic heterogeneity, the latter with the presence of dominant sublineages with epidemic behavior, which can acquire functional advantages over other strains in their ability to transmit and cause disease (Coscolla and Gagneux, 2014; Folkvardsen et al., 2018). In Colombia, lineage-4 was found as the most predominant. A previous molecular epidemiology study showed the population structure of *Mtb* in certain geographic regions of the country with a dominance of the LAM and Haarlem sublineages, particularly in Medellín and Cali (Realpe et al., 2014). This study found that in North-Eastern (NE) area of Medellín the sublineages LAM9 SIT42, H1 SIT62 predominated, compared with other sublineages present in the same geographical area. However, there is still limited information about the genetic background of *Mtb* associated with the high or low prevalence of clinical isolates, which generates the need to increase knowledge in this field that contribute to the control of the *Mtb* transmission (Folkvardsen et al., 2018).

Due to the development of whole-genome sequencing (WGS), it is possible to perform comparative genomic analysis of several strains. These have now become an alternative in order to answer microbiological questions related to outbreaks, evolution, antibiotic resistance, pathogenicity, and transmission (Uchiya et al., 2017). Analyses of multiple genomes of individuals from the same species have revealed wide intra-species diversity, largely due to differences in the gene and transposable element repertoire of the strains (Tettelin et al., 2008). Here, we first explore the *Mtb* pan-genome by estimating the genomic diversity of 47 *Mtb* clinical isolates of lineage-4. This was done by identifying genes shared among all isolates under study (core genome), and genes present in some but not all the strains studied (dispensable genome), as well as the strain-specific genes (Tettelin et al., 2005). In addition, we report a pan-genome-wide association study (pan-GWAS) where genetic variants related to the phenotype of the high or low prevalence of *Mtb* clinical isolates were identified.

## Materials and methods

### Study population

*Mycobacterium tuberculosis* strains were isolated from patients with pulmonary TB in the laboratory of Corporación para Investigaciones Biológicas as part of the Colombian center for TB research developed in Colombia between 2005 and 2008. A total of 324 isolates were genotyped by spoligotyping (Spoligo-International-Type—SIT) and 24-loci Mycobacterial Interspersed Repetitive Units-Variable-Number of Tandem Repeats (MIRU-VNTR). Out of all typed isolates, 135 (41.7%) of them were present in the NE area of Medellín that has shown the highest incidence in the city per 100,000 inhabitants (81 in 2018), with an incidence average rate of 70 per 100,000 in last decade (Secretaría Seccional de Salud y Protección Social de Antioquia, 2017; Almanza et al., 2019). *Mtb* LAM9 SIT42 and Haarlem1 SIT62 sublineages had the highest prevalence (73.3%) compared with other sublineages that exhibit lower prevalence (less than 10%). Simple random sampling with Epidat v4.1 was performed on the different genotypes, selecting 27 high prevalence and 20 low prevalence isolates for this study (Table 1).

**TABLE 1:** *Mycobacterium tuberculosis* (*Mtb*) isolates recovered in the city of Medellín between 2005 and 2006.

Selected isolates in Medellín (324)						
North-Eastern area (135)					Selected isolates	
Clade	SIT	N. isolates	Isolates (%)	Prevalence	SRS*	Isolates 48
LAM9	42	49	36.30%	High	11	27
H1	62	50	37.00%	High	16	
H3	Diff* (4)	11	<8.1%	Low	9	20
H1	Diff* (6)	11	<8.1%	Low	6	
LAM	Diff* (11)	14	<10.4%	Low	5	

The 41.7% were recovered in the NE area of Medellín. The word Diff* corresponds to the number of isolates with different SIT and SRS* corresponds to a simple random sample using Epidat v4.1.

### DNA isolation and whole genome sequencing

All 47 *Mtb* isolates were grown in Middlebrook 7H11 medium at 37°C in a 5% CO2 atmosphere for 3 weeks. DNA isolation was performed as previously described (van Soolingen et al., 1994). In brief, three loopfuls of cells were suspended in 400 ml of 1× Tris-EDTA buffer 1X (10 mM Tris–HCl pH 8, 0, 1 mM EDTA). Heat inactivated at 80°C for 45 min. Then, 50 μL of lysozyme (10 mg/ml) was added, and incubated for 15 min at 37°C. Then, 75 μl of 10% SDS and 6 μl proteinase K (10 mg/ml) was added and incubated 10 min at 65°C. Followed by the addition of 100 μL CTAB (cetyltrimethylammonium bromide) 10% and NaCl al 5% preheated solution at 65°C, vortexed and incubated at 10 min at 65°C. Protein/lipids separation was performed with 750 μL chloroform/isoamyl alcohol (24: 1, v/v) vortexed, and centrifuge for 8 min at 12,000 g. The supernatant was transferred to a 1.5 μl tube and 450 μL of 2-propanol was added for to nucleic acid precipitation at −20°C for 30 min. After centrifugation, the pellet was washed with 70% EtOH and subsequently air-dried and re-suspended in 20 μl Tris-EDTA (TE) buffer at 37°C. Genomic DNA of the 47 *Mtb* isolates was sequenced at the Biotechnology Center of the University of Wisconsin using Illumina HiSeq 2500 (150, paired-end) with TruSeq Nano library prep according to the manufacturer protocol (Illumina, CA, USA).

### Bioinformatic analysis: Pre-processing, *de novo* assembly, and annotation

Quality control of the sequencing raw data was performed with FastQC 0.11.3 (Andrews, 2010). The pre-processing tool Trimmomatic v3 (Bolger et al., 2014) was used to remove adapters, trimmed bases by position, clean artifacts and keep a minimum quality threshold Q22. *De novo* assembly of mycobacterial genomes was performed with SPAdes v3.8.2 (Bankevich et al., 2012) using different odd *k*-mer sizes in the range *k* = 21 to *k* = 87. Assembly stats such as statistics such as N50, largest contig, GC-content, genome fraction covered, and genes number were computed with Quast v3.1 (Gurevich et al., 2013) to assess the quality and compare it to the reference genome H37Rv (NC_000962.3). In addition, correction of assembly errors was carried out using Pilon v1.22 (Walker et al., 2014). To call features within contigs, genome annotation was performed using Prokka v1.11 (Seemann, 2014), with protein-coding genes predicted with Prodigal (Hyatt et al., 2010) and homology searches done against UniProt (Swiss-prot) database (Apweiler, 2004) using BLASTP (Altschul et al., 1990) with an *E*-value of 1e-6.

### Pan-genome construction, core and accessory genome evolution

Identification of clusters of homologous genes within the 47 genomes annotated was performed with GET_HOMOLOGUES V09062017 (Contreras-Moreira and Vinuesa, 2013), using BLASTP with a sequence identity of 90% and minimum default query coverage of 75% for paired alignments. Once the local alignments were sorted and indexed, three algorithms, Bidirectional best-hit (BDBH) (Contreras-Moreira and Vinuesa, 2013), OrthoMCL (OMCL) (Fischer et al., 2011), COGtriangles (COG) (Kristensen et al., 2010) also included in GET_HOMOLOGUES software, were used to produce sequence clusters (both nucleotide and peptides) according to protein sequence similarity. Single-copy orthologous clusters were defined as those containing only one sequence from each input genome; extra copies were considered paralogous.

To estimate the core genome size, a genome composition analysis was done using the *get_homologues.pl –c* script. Syntenic groups of genes were identified at the intersection of the three clustering algorithms to calculate the overlap of orthologous protein sets. Accordingly, we estimated the size of the intersected core genome, defined as the subset of clusters with genes from all genomes. Similarly, the intersection of the COG and OMLC algorithms generated a binary matrix in tabular format that summarized genes present (1) and absent (0) in each of the 47 genomes. This matrix represents the pan-genome, the complete repertoire of genes of the 47 *Mtb* genomes lineage-4.

In order to measure how much the core and the accessory genomes of the *Mtb* Lineage-4 change as new isolates are added, a genome composition analysis was performed. This is a simulation that estimates how many core and novel sequences are added by genomes sampled in random order. This was done after taking 10 replicates using random permutations of the 47 strains using the *get_homologues.pl* and *plot_pancore_matrix.pl* scripts, producing plots with the fitted functions proposed by Tettelin (Tettelin et al., 2005).

### Functional pan-genome analysis and pan-genome wide association study of *Mtb* lineage-4

The data of the pan-genome matrix (PGM) and the GeneBank annotation files were used as input in BPGA v1.3 software (Chaudhari et al., 2016). Identifiers with the best hits were assigned from the KEGG (Kyoto Encyclopedia of Genes and Genomes) and COG (Clusters of Orthologous Groups of proteins) reference databases (Kanehisa and Goto, 2000; Tatusov et al., 2000), for which USEARCH’s ublast (Edgar, 2010) function was used, with an *E*-value cutoff of 10E-5, databases and tools were used by default in BPGA V1.3. This analysis allowed us to annotate metabolic pathways and the functional categories of COGs within the dispensable genome, the core genome, and the unique genes that make up the pan-genome.

An association study was conducted based on patterns of gene presence/absence in the pan-genome. The polymorphisms considered for analysis included changes caused by the presence of variants such as those containing insertions or deletions, correlating them with the characteristics of high or low prevalence of *Mtb* strains. The PGM containing the variants and a matrix with the prevalence trait were used such as input for Scoary v1.6.16 (Brynildsrud et al., 2016). The observed presence/absence was correlated with the prevalence by evaluating its significance through Fischer’s exact test. A list of variants with a *P*-value < 0.05 was generated. To avoid false results as significant and avoid the probability of the family wise error rate (FWER), an adjustment of the *P*-value with tests of Bonferroni’s and Benjamini–Hochberg correction was performed.

The insertions, deletions or genes previously identified and significantly associated with high and low prevalence of *Mtb* strains were individually verified by multiple genome alignment with Mauve v2.4.0 (Darling et al., 2004). This software can also generate a file in tabular format with the SNPs and its coordinates in each genome. This can be used to identify differential SNPs between high and low prevalence strains. Gaps in the alignments were removed, and minor nucleotide frequency in each position computed with a script available at https://github.com/ualonso85/Thesis-pipeline/blob/master/freqs.sh.

To assess the accuracy of our sequencing and bioinformatic approaches, we amplified the sequence region of the variants associated with the high or low prevalence in 18 *Mtb* strains randomly selected. The PCR products were sequenced on an ABI 3730 sequencer (Applied Biosystems). There was 100% identity between all sequenced regions both by Illumina HiSeq 2500 and ABI 3730.

### Phylogenomic analysis

The PGM generated from the consensus of orthologs groups with the COG and OMCL algorithms was used for the phylogenomic reconstruction of the 47 genomes. The phylogenetic trees were built with the parsimony method from discrete characters (present/absence genes) using the PARS v3.69 software include in PHYLIP suite. In addition, a maximum likelihood method was used for phylogenomic analysis with the GTR substitution model and ultrafast bootstrap of 1000 replicates (–bb 1000 -alrt 1000) using IQ-TREE v1.6.12 (Nguyen et al., 2015). The signal of each genetic tree was calculated based on the mean values of branch support with the SH-aLRT and UFBoot tests. The grouping of clades in the phylogenomic tree was verified with 26 *Mtb* genomes of different lineages (L2, L3, L4, L6) with known tree topology downloaded from the NCBI database.

Phylogenomic reconstruction with SNPs was performed by converting the eXtended Multi-Fasta (XMFA) alignment file from Mauve to fasta format with a script available at https://github.com/eead-csic-compbio/eead-csic-compbio.github.io/blob/master/scripts/xmfa2fasta.pl. Trimal software v1.2rev59 (Capella-Gutierrez et al., 2009) was used to removed gaps leaving only the nucleotide positions that were present in all *Mtb* genomes (core). The concatenated sequences from each genome that contained the SNPs were used to build the phylogeny using the maximum likelihood method with the same parameters in IQ-TREE described above but using TVM (transversional model) such as nucleotide substitution model. A strain of *Mycobacterium canettii* 140010059 Accession NC_015848 was included as root.

### Data availability

The data generated during the current study are available online at National Center for Biotechnology Information under Bioproject accession PRJNA838941.

### Ethics statement

This study was approved and carried out in accordance with guidelines and regulations by the Ethics Review Committee of Coporación para Investigaciones Biológicas.

## Results

### Prevalence of *Mtb* lineage- 4 in NE area of Medellín

In the Medellín city, 135 *Mtb* isolates were recovered from Popular, Santa Cruz, Manrique, and Aranjuez communes, from 2005 to 2006. The spoligotype of these isolates were represented by patterns of presence or absence of some of the 43 spacers in the Direct Repeat (DR) locus. Sublineage classification was performed by assessing the SIT code retrieved from the SPOLDB4 database, as well as with 24 MIRU-VNTR analyzed online with MIRU-VNTR *plus* tool (Supplementary Table 1). In total 99 (73.3%) of the 135 isolates were classified as high prevalence due to the high frequency of the Haarlem1 SIT62 and LAM9 SIT42 sublineages, with a relative frequency of 0.37 and 0.36, respectively. The remaining 26.7% corresponded to 34 isolates of different sublineages with low frequency (<0.10) in the same geographical area and during the same period. Simple random sampling allowed the selection of 27 high-prevalence isolates and 20 low-prevalence isolates that represented the largest number of genotyped sublineages (Supplementary Table 2).

### *M. tuberculosis* isolates and general genomic features

Once the 47 *Mtb* isolates were defined according to the high or low prevalence, each isolate was sequenced. A total of 156,746,390 reads of the isolates were obtained after filtering by quality parameters, with an average of 3,335,030 reads per sample. The average genome size of the 47 isolates was 4.348 Mb, G + C 65.5%, genome fraction 98.6%, with coverage depth of 102X (ranging from 30X in UT331 to 198X in UT86), and N50 was 114,855 (ranging from 78,270 in UT414 to 166,979 in UT86) with 105 contigs on average (ranging from 66 in UT105 to 140 in UT487) (Supplementary Table 3). Where possible, assembly errors were corrected by remapping reads, which considers alignment differences and reads coverage for each genome. Isolates UT487 and UT30 were taken as an example to show the most identified types of assembly errors in the data set (Supplementary Table 4).

Regarding gene model annotation, the UT53 genome presented the lowest number with 4,024 CDS, and UT08 presented the highest number with 4,087 CDS. An average of 2,880 (71%) CDS corresponded to proteins where function can potentially be assigned by homology; furthermore, 1,122 hypothetical proteins (28%), and 52.9 tRNAs (1%) were annotated in the 47 genomes (Supplementary Figure 1).

### Pan-genome and core-genome of *Mycobacterium tuberculosis* lineage-4

Comparative analysis allowed us to determine the complete repertoire of genes of the 47 *Mtb* lineage-4 genomes, that together make up the pan-genome. The core-genome was composed of gene clusters shared among all isolates representing 73.5% of the pan-genome resulting from the intersection of the COG, BDBH and OMCL algorithms (see section “Materials and methods” and Supplementary Figure 2). In addition to the core subset, the accessory genome containing the dispensable genes and the strain-specific genes together added a total of 4,846 gene clusters (Supplementary Figure 3).

**FIGURE 1:**
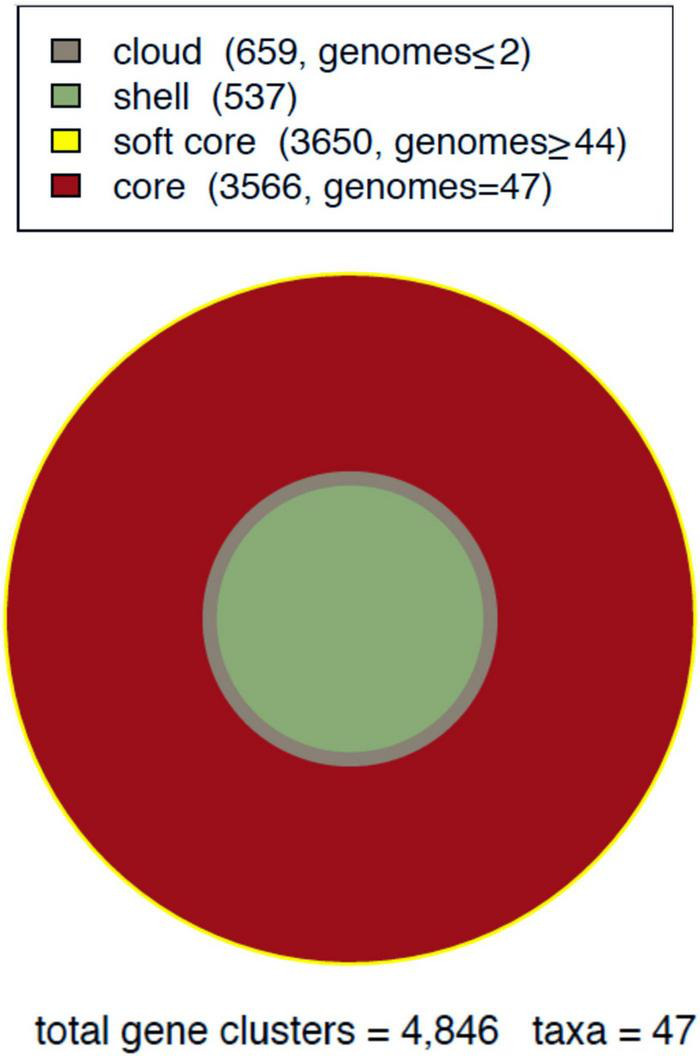
*Mycobacterium tuberculosis* (*Mtb*) pan-genome area of lineage-4. Global composition of gene clusters divided into four compartments.

The complete repertoire of 4,846 gene clusters of the pan-genome was classified into four occupancy classes, as summarized in Figure 1 (Contreras-Moreira and Vinuesa, 2013):

1) Core: genes conserved in all isolates studied.
2) Soft core: genes found in 45 (95%) or more of the isolates, thus including core genes.
3) Shell: Gene clusters conserved in a variable number (3 to 44) isolates.
4) Cloud: rare or unique genes present in two or fewer isolates.

**FIGURE 2:**
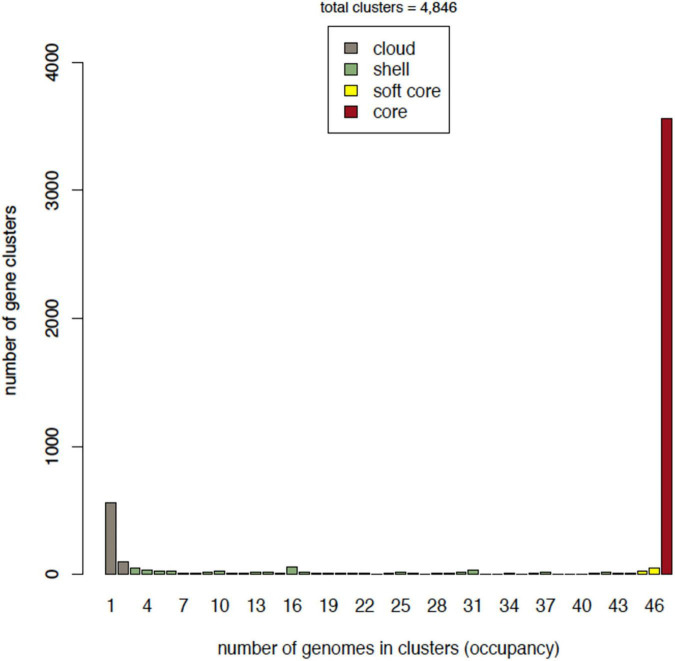
Occupancy of genomes in gene clusters. Every compartment or class is depicted in a different color. The *X*-axis shows the occupancy, which is the number of genomes that are contained in a given number of clusters of genes.

Accordingly, the core compartment has the highest occupancy, and includes 3,566 gene clusters. The soft-core class amounts to a total of 3,650 (75%) gene clusters, which includes the strict core. The shell class contained 537 gene clusters, representing 11% of the total. Finally, cloud clusters represented 13% of the pan-genome (Figure 2).

**FIGURE 3:**
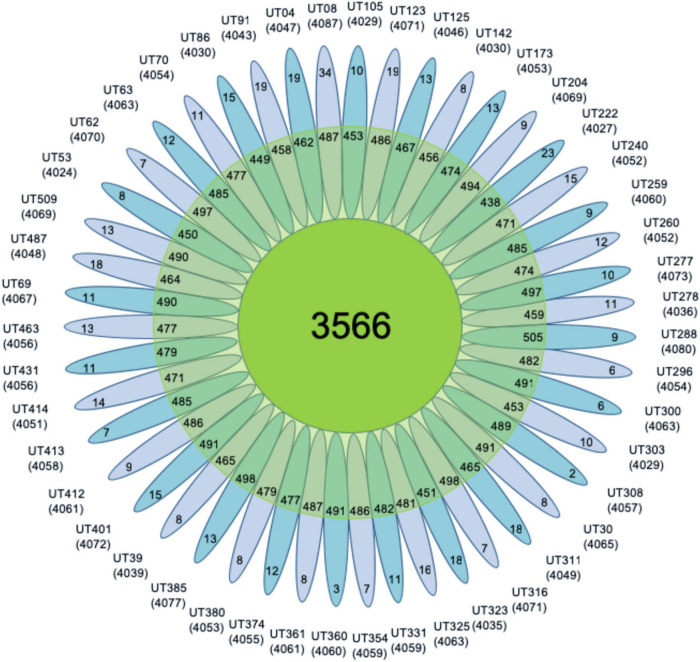
Pan-genome flower. It shows all *Mtb* lineage-4 isolates that make up the pan-genome. In the center the number of core genes is observed, the second clear circle shows the accessory genes, and the petals show the number of specific genes of each isolate in the 47 genomes. The numbers below each isolate denote the total number of related CDSs.

**FIGURE 4:**
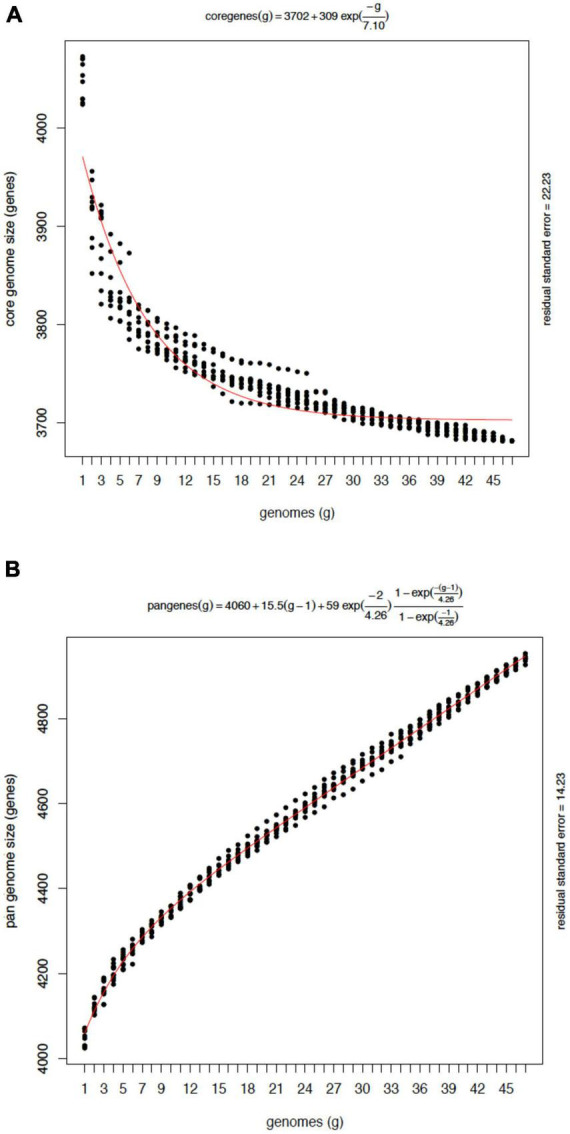
Composition analysis of the core and the pan-genome of *Mtb* L4 from 47 genomes. **(A)** Each point on the *Y*-axis indicates the number of gene clusters after adding a new genome in randomized simulations. The line red indicates the exponential decay as a function of the average values of the clusters each time a genome was added to the analysis. **(B)** Pan-genome growth simulation by counting new genes added by the last genome sampled. Note that sequences matching a previously seen gene coverage ≥20% will be considered homologous and thus won’t be considered new. An open pan genome model is observed.

On average, the accessory genome consisted of 477 genes with a standard deviation (SD) of 16, with 12 strain-specific genes (SD = 5.5). The average number of CDS in each isolate annotated was 4,055 with an SD of 15 (Figure 3). Moreover, to assess whether the number of sequenced genomes was sufficient to describe the content of the core and accessory genome of the *Mtb* lineage-4, a genome composition analysis was performed. Both in the core and in the accessory genome, a change was observed as a function of its size each time a genome was added in random order until the 47 genomes were completed. The evolution of core and accessory genome size was analyzed in terms of exponential decay and growth models. The fitted exponential decay suggested that the number of orthologous gene clusters in the core tends to plateau near 3,700 gene clusters (Figure 4A). Conversely, the exponential growth model fails to plateau in our simulation and continued to grow above 4,900 gene clusters (Figure 4B). The number of non-redundant genes found in *Mtb* isolates would seem to increase by approximately 15 genes each time a new genome is added.

### Functional pan-genome analysis

A functional profile of the core-genome was obtained by annotating COG categories. The [R] general function was the most frequently observed (16%), followed by [I] Lipid transport and metabolism (8%), [S] Function unknown (8%), [Q] Secondary metabolites biosynthesis, transport and catabolism (7%), [K] Transcription (7%), [C] Energy production and conversion (7%), [E] Amino acid transport and metabolism (7%), and [L] Replication, recombination and repair (5%). Other functional categories were found related to processes of the central metabolism of mycobacteria such as translation, transport biogenesis and metabolism of carbohydrates and coenzymes, among others (Supplementary Figure 4).

**FIGURE 5:**
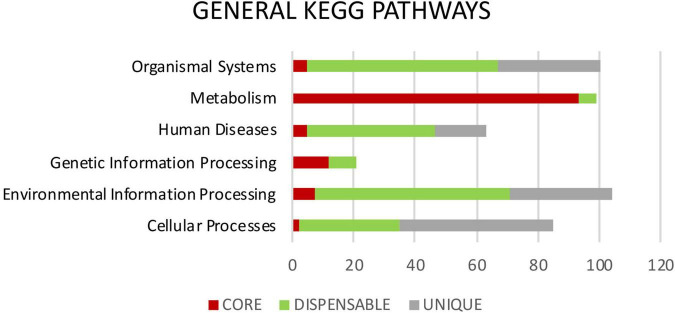
General KEGG pathways. It shows the differences in percentage of the functional annotations at the highest hierarchical level between the genes highly conserved of the core (red), genes moderately conserved of the dispensable genome (green) and genes exclusive to each isolate or unique (gray).

**FIGURE 6:**
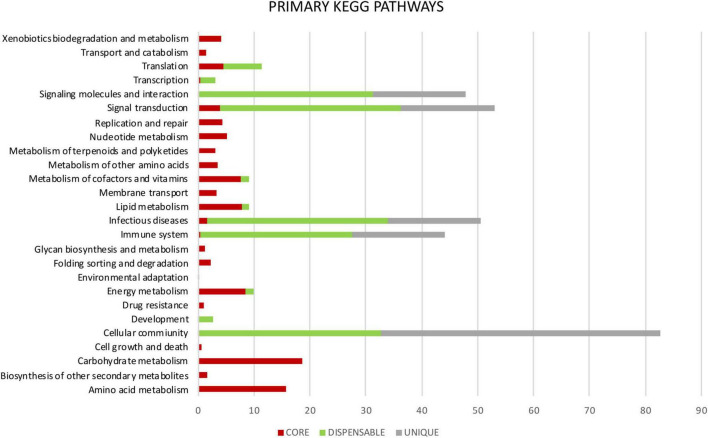
Primary KEGG pathways. It shows the differences in percentage of the functional annotations among the genes highly conserved of the core (red), where the highest number of assignments for each category is observed. Genes moderately conserved of the dispensable genome (green) are mainly related to adaptation to the environment-host and genes exclusive to each isolate or unique (gray).

Metabolic pathways were identified in *Mtb* pan-genome using the KEGG databases. The highest hierarchical levels found for core gene clusters were related to metabolism (75%); many of them with the metabolism of carbohydrates (18%), lipids (8%), amino acids (16%), cofactors and vitamins (8%), and the remaining 25% associated with replication, repair, translation and signal transduction among others (Figures 5, 6). The general functions of the dispensable genome in the KEGG pathways were related to environmental information processing (30%), primary paths of cellular community (19%), signal transduction (19%) and signaling molecules and interaction (18%). Among general pathways, genes were found to be associated with organismal systems (29%) and human diseases (19%). The majority of those were assigned to primary functions such as the immune system (16%) and infectious diseases (19%) (Figures 5, 6). For the case of strain-specific genes, cellular processes were the general function with the highest assignment (38%), followed by organismal systems (25%), environmental information processing (25%), and human diseases (13%) (Figure 5).

### Pan-genome association study of *Mtb* lineage-4

The prevalence phenotype was correlated with the presence or absence of genes in the pan-genome. Variants such as insertions and deletions were identified in gene regions related to their presence or absence. Each variant was initially assigned the null hypothesis of no association with prevalence. A total of 115 variants in the 47 genomes of *Mtb* lineage-4 were found to be associated to the trait of high or low prevalence (*p*-value < 0.05). Nevertheless, due to the high number of null hypotheses evaluated and the multiple factors analyzed simultaneously in the pan-GWAS, *p*-values were adjusted with the Bonferroni’s method, as implemented in Scoary. With this correction in place, four genes were significantly associated (*mmpl12*, *PPE29*, *Rv1419*, *Rv1762c*). Due to the conservative nature of the Bonferroni’s multi-testing correction, the Benjamini-Hochberg correction (FDR) was also considered. In this case, the list of associated genes comprised seven additional genes: *Rv3371*, *Rv2735c*, *scoA*, *mhpE*, *PE-PGRS52*, *lppB*, and *gabD2* (Table 2). The name of each isolate, its genotype, as well as the number of isolates associated with high or low prevalence are shown in Supplementary Table 5.

**TABLE 2:** List of *Mtb* lineage-4 genes identified in pan-GWAS analysis.

Gene	Uniprot code	Functional annotation	*P*-value	Bonferroni’s-adjusted *P*-value	Benjamini-H. adjusted *P*-value	Variant association
*mmpL12*	A0A0T9BXN4	Probable transport of membrane. Responds to host immune response.	1.13e-07	0.00015	2.90e-05	Low prevalence
*PPE29*	P9WI09	May be required for invasion of host endothelial cells	1.13e-07	0.00015	2.90e-05	Low prevalence
*Rv1419*	P9WLX8	Uncharacterized protein/GO carbohydrate binding	5.51e-07	0.00071	7.84e-05	Low prevalence
*Rv1762c*	A0A1K6RHD7	Domain of uncharacterized function (DUF74)/GO transmembrane	5.51e-07	0.00071	7.84e-05	Low prevalence
*Rv3371*	P9WKA9	Putative diacylglycerol O-acyltransferase	0.00014	0.18124	0.0062	High prevalence
*Rv2735c*	R4ML16	hypothetical protein uncharacterized	0.00014	0.18124	0.0062	High prevalence
*scoA*	P9WPW5	Probable succinyl-CoA:3-ketoacid coenzyme A transferase subunit A	0.00014	0.18124	0.0062	High prevalence
*mhpE*	R4MB83	4-hydroxy-2-oxovalerate/4-hydroxy-2-oxopentanoic acid aldolase	0.00014	0.18124	0.0062	High prevalence
*PE-PGRS42*	I6XEF1	Family PE-PGRS proteins	0.00014	0.18124	0.0062	High prevalence
*lppB*	P9WK79	Putative lipoprotein LppB, extracellular region/GO cell membrane	0.00014	0.18124	0.0062	High prevalence
*gabD2*	P9WNX7	Putative succinate-semialdehyde dehydrogenase [NADP(+)] 2	0.00014	0.18124	0.0062	High prevalence

### Identification of indels and SNPs associated with the prevalence

Multiple alignments of the 47 genomic isolates plus the *Mtb* reference genome H37Rv allowed us to verify the differential genetic variants between the high and low prevalence isolates. For instance, we discovered that the *mmpL12* gene locus, a single CDS associated with high-prevalence, turned out to be split into two neighbor CDS by the IS6110 insertion in low-prevalence genomes. This type of insertions generally results in inactivation corresponding gene (see Supplementary Figure 5; Reyes et al., 2012). Overall, 5 variants significantly associated to prevalence traits corresponded to insertions: in the *mmpL12* gene between position 1642_3000ins, *PPE29* between position 639_641insG, *Rv2735c* position 363_364insGT, *ScoA* position 476_478insG, *gabD2* between position 610_644ins. Moreover, 3 deletions were also identified: in the *Rv1419* gene position 199delA, the *Rv3371* gene position 376delA and the *PEPGRS42* gene between positions 1382_1417del (see Table 3).

**TABLE 3:** Genetic variants associated with high or low prevalence of clinical isolates of *Mtb* lineage-4 in the North-Eastern zone of Medellín.

No.	Gene	Size (pb)	Locus (pb)	Variant	Genomes number high prev. *N* = 27	Genomes number low prev. *N* = 20	Benjamini-H. adjusted *P*-value	Variant association
1	*mmpL12*	3.341	1.714.172	1642_3000ins	0	14	2.90e-05	Low prevalence
2	*PPE29*	1.272	2.042.001	639_641insG	0	14	2.90e-05	Low prevalence
3	*Rv1419*	474	1.593.505	199delA	0	14	7.84e-05	Low prevalence
4	*Rv1762c*	789	1.995.054	538C > T	0	13	7.84e-05	Low prevalence
5	*Rv3371*	1.341	3.784.932	374T > A, 376delA	16	1	0.0062	High prevalence
6	*Rv2735c*	993	3.047.560	363_364insGT	16	1	0.0062	High prevalence
7	*scoA*	747	2.819.124	476_478insG	16	1	0.0062	High prevalence
8	*mhpE*	1.011	3.886.073	169G > T	16	1	0.0062	High prevalence
9	*PE-PGRS42*	2.085	2.795.301	1382_1417del	16	1	0.0062	High prevalence
10	*lppB*	561	2.867.124	362C > A	16	1	0.0062	High prevalence
11	*gabD2*	1.557	1.957.577	610_644ins	16	1	0.0062	High prevalence

Single nucleotide polymorphisms (SNP) identified in the multiple alignment of the 47 *Mtb* genomes were obtained taking as reference the H37Rv genome. This was done after obtaining the coordinates of the SNPs in each of the genome alignments. Significantly associated SNPs (*p*-value < 0.05 Benjamini-H correction) were identified in several genes: *Rv1419* positions 122G > A, 362C > A, *Rv1762* position 538C > T, *Rv3371* positions; 374T > A, *mhpE* position 169G > T, and in the *lppB* gene position 362C > A (see Table 3).

All in all, a total of 12 genomic variants were identified combining indels and SNPs. These are distributed in 11 CDS. Each of these genetic loci were amplified by PCR to confirm our findings. Eleven of the 12 genetic loci were successfully amplified with sizes ranging from 278 bp for the *RV2735c* gene to 1650 bp for the *mmpL12* gene (Supplementary Table 6); the *PE-PGRS42* gene did not amplify despite using two different pairs of primers. PCR products sequenced on an ABI 3730 had 100% identity among all sequenced regions when compared to the Illumina HiSeq 2500 sequence.

### Phylogenomic analysis of the *Mtb* lineage-4

**FIGURE 7:**
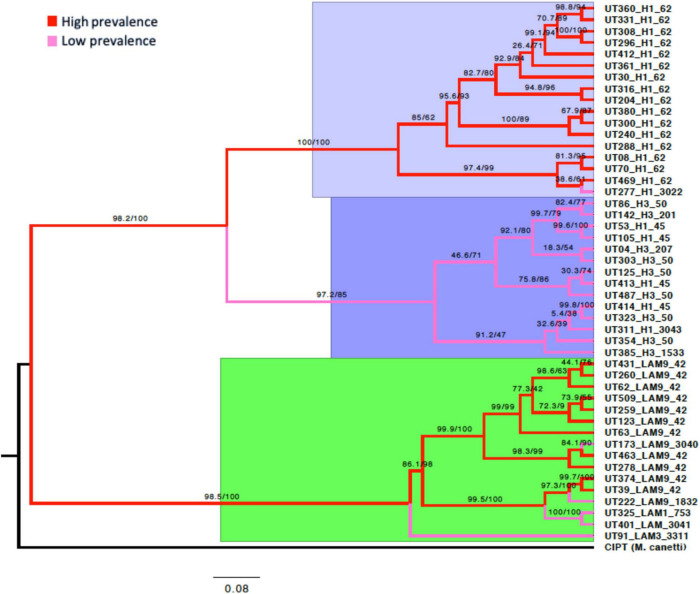
Phylogeny of the *Mtb* L4 pan-genome by maximum likelihood. IQ-TREE was used to estimate the phylogenetic tree from the consensus of the 4,846 gene clusters produced by both the COG and OMCL algorithms. The node supports after 1000 bootstraps is shown on the branches. The outgroup is *M. canetti*. Three major clades were observed. Lilac; all the branches of the isolates, except UT277, coincide with the Haarlem1-SIT62 sublineage considered to be of high prevalence. Purple; the branches correspond mainly to Haarlem1-SIT45 and Haarlem3-SIT50 isolates, and less frequently to Haarlem1 and Haarlem3 with variable SIT. Green; a clade was observed mixed with branches of isolates considered to be of high prevalence, mostly LAM9 SIT42, and four branches of low prevalence belonging to the LAM sublineages. The red branches correspond to isolates with high prevalence and the pink branches correspond to all isolates with low prevalence in the North-Eastern zone of Medellín.

The analysis of the discrete characters of the PGM allowed the phylogenomic reconstruction of the isolates of *Mtb*, capturing the phylogeny implicit in the matrix. The tree showed high branch support values. However, some branches in each of the three main clades showed support values lower than 70% (see Figure 7). The tree was rooted with the *Mycobacterium canetti* species that was used as an “outgroup” genome. The topologies suggest three main clades that represent the evolutionary relationships of the 47 *Mtb* genomes based on their gene content (Figure 7).

As the phylogenomic reconstruction was conducted only with genomes of isolates that belonged to the *Mtb* lineage-4, we wanted to confirm whether the topology of the branches was conserved when adding to the analysis 26 complete *Mtb* genomes from different lineage [11 lineage-4 (Euro-American), 8 lineage-2 (East-Asian), 4 Lineage-3 (East-African-Indian) y 3 Lineage-6 (West-Africa)] previously downloaded from NCBI database. A total of 6,160 clusters of protein group were obtained and computed from the PGM. The topology obtained was consistent with the trees obtained by maximum likelihood of lineage-4, in addition to coinciding with the topology of the trees established as a reference for the grouping of these lineages (Ngabonziza et al., 2020), coinciding with the most accepted phylogenetic history for this species. This allowed us to observe that the topology obtained based on the genetic content was adequate and can be used as a reference in future comparisons (Supplementary Figure 6).

**FIGURE 8:**
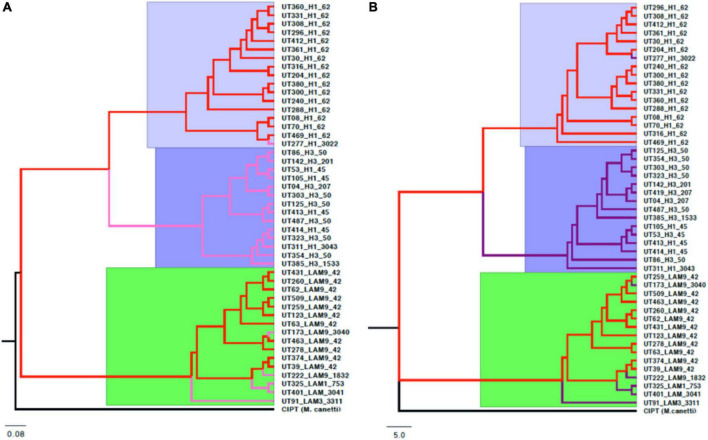
Topology comparison trees of Mtb L4 were constructed by maximum likelihood using different molecular markers. IQ-TREE was used to estimate the phylogeny with node supports after a bootstrap of 1000 replicas. **(A)** Consensus of gene clusters of the pan-genome (4,846 genes). **(B)** Concatenation of 34,999 SNPs of the core genome. In both, three main clades were observed, with very similar topologies and distribution of the isolates in each of the branches, despite having used different markers as an approximation.

From 34,999 SNPs present in the core genome, the reconstruction of phylogenetic trees was carried out with CIPT *Mycobacterium canetti* strain, which was used as root. The support values were greater than 95% for most of the branches of the tree, however, some of the branches presented support values lower than 70% (Supplementary Figure 7). The topologies showed the same three clades that represented the evolutionary relationships of the 47 *Mtb* genomes in the pan-genome phylogeny described above, retaining a similar distribution of isolates in each branch of the tree (Figure 8). Both approaches also match the classification of the sublineages reported by molecular genotyping methods (Spoligotyping and 24-loci MIRU-VNTR).

## Discussion

### *Mtb* pan-genome and their accessory genes

This is the first pan-genomic analysis of *Mtb* lineage-4 (Euro-American) carried out in Colombia, characterized by being the most dominant lineage in the country. The pan-genome estimation showed that the number of genes with new characteristics continues to increase regardless of the number of *Mtb* genomes added to the analysis. Experimental results and predictions with mathematical models have shown that in some species new genes are discovered even after the sequencing of hundreds of genomes for a particular species (Medini et al., 2005; Rouli et al., 2015). The size of the pan-genome is related to the events of loss or gain of genes. If the niche changes, some functions might be used less and eventually lost. Unlike bacteria that are found in diverse environments, which frequently undergo gene gain events, bacteria that have a lifestyle restricted to a specific niche possess a smaller and specialized genome, therefore the pan-genome tends to be closed or finite (Medini et al., 2005; Rouli et al., 2015). However, our results showed that *Mtb* lineage-4 in the NE area of Medellín has an open pan-genome, this may be partly related to the low number of genomes used in this study. We found a great genetic variability despite this species being restricted to a specific niche with a more conserved genome (Medini et al., 2005; Yang et al., 2018; Woodman et al., 2019). Therefore, intra-species diversity at the sublineage level was broader than expected. Recent composition analysis studies of the *Mtb* pan-genome have shown that by increasing the number of genomes above 120, the curve tends to flatten considering the pan-genome of this species is almost closed. Nevertheless, it continues to present a significant number of variations (Dar et al., 2020; Zakham et al., 2021).

The pan-genome of *Mtb* lineage-4 was rather associated with the presence of smaller indels and SNPs that cause frameshifts or premature stop codons in genes increasing the genetic variability. Nevertheless, this variability is not as abundant as compared to other bacterial species where gene gain events are common due to recombination mechanisms (Rouli et al., 2015). These results suggest that despite the fact that *Mtb* does not have horizontal gene transfer, it has mechanisms for genetic variability such as transposable elements and a high frequency of mutations in a latency state (microevolution) (Coros et al., 2008; Vernikos et al., 2015).

Out of the identified 4,846 genes in the pan-genome 1,071 (26.5%) corresponded to hypothetical proteins representing similar proportion to the one reported previously in *Mtb* (Cole et al., 1998). A total of 1,196 genes were identified in the accessory genome; composed of dispensable genes located in the shell compartment and unique genes located in the cloud. Of the 537 genes in the shell, 99 coded for PE family genes including 22 PE-PGRS genes and 17 for PPE family genes. The PE/PPE family represents 10% of the genes encoding *Mtb* and its proteins present the greatest source of antigenic variability between different isolates, it is assumed that they are of critical importance in the general pathogenesis of TB (Mukhopadhyay and Balaji, 2011).

In the shell compartment, 237 genes encoded hypothetical proteins representing 44% of the genes in this compartment, and are responsible for much of the genetic variability that remains unknown (Mukhopadhyay and Balaji, 2011; Vernikos et al., 2015). These dispensable genes shared between certain groups of isolates contribute to diversity, and presumably play roles in complementary biochemical pathways not essential for growth. However, they may confer selective advantages and adaptation to different niches, new host colonization, and antibiotic resistance (Vernikos et al., 2015). For instance, the Beijing genotype of lineage-2 has been characterized by having a higher transmission frequency than other lineages, possibly due to shell genes, in addition to being associated with a higher drug resistance, which suggest a relatively high transmission fitness (Holt et al., 2018; Nieto Ramirez et al., 2020).

Of the 659 strain-specific genes located in the cloud compartment, 274 coded for hypothetical proteins, 224 of them belonging to the PE family of which 53 coded for proteins of the PE-PGRS family, representing together 83.6% of the genes of the cloud. Genes in the PE/PE-PGRS family are known to have multiple copies of polymorphic repetitive sequences; however, this high variability added to the lower quality contigs and scaffolds assembled for these regions, could cause this overestimation (Mukhopadhyay and Balaji, 2011; Periwal et al., 2015).

### Functional analysis of the accessory *Mtb* genome

Among the COG categories assigned to the 537 genes that were part of the dispensable genome, four had the highest proportion of genes related: Q] Secondary metabolites biosynthesis, transport and catabolism, followed by [D] Cell cycle control, cell division, chromosome partitioning, [U] Intracellular trafficking, secretion and vesicular transport and [S] Function unknown. Many of these genes are partially shared between *Mtb* isolates and may represent particular metabolic characteristics associated to adaptation to one human host or another. Most of the dispensable genes were found to be related to general KEGG pathways for Environmental Information Processing and Human Diseases that causes in the host (Figure 5 and Supplementary Figure 4). The presence or absence of these genes could be the result of adaptation processes by *Mtb* to the host or selection pressure exerted by the environment (Medini et al., 2005; Vernikos et al., 2015). The dispensable genes, despite being secondary genes, are important for adaptation, they code for important functions related to virulence and pathogenicity (Contreras-Moreira and Vinuesa, 2013). Differences in these genes make it possible to distinguish between lineages. Modern lineages such as lineages 2 and 4, have been characterized by being more virulent and transmitted more successfully within the global population than other lineages (Coscolla and Gagneux, 2014). Our data confirm that the high resolution of whole genome sequencing has not only allowed the differentiation between different lineages, but also between sublineages present in the same geographic population.

Among strain-specific genes, they were mostly enriched in COG categories related to [R] general function prediction and [U] Intracellular trafficking, secretion and vesicular transport, follow by [M] Cell wall/membrane/envelope biogenesis, [T] Signal transduction mechanisms and by [Q] Secondary metabolites biosynthesis, transport and catabolism. Among these functions, the transport and secretion of vesicles by *Mtb* through their membrane and cell wall has been described to have an important role in the host interaction (Gupta and Rodriguez, 2018). The release of extracellular vesicles allows the interaction of the cell with the environment. These vesicles in pathogenic bacteria play roles in cell-cell communication, immunomodulation, virulence, and cell survival (Gupta and Rodriguez, 2018). It is possible that some of these strain-specific genes in each isolate give it unique characteristics to interact individually with its host, allowing it to adapt to changing external conditions.

### The *Mtb* core genome: Analysis and functional classification

The core represented the largest fraction of the pan-genome, including more than 70% of the genes identified. As expected, every time a new genome was added to the analysis, the size of the core genome decreased. Many of these genes are constitutive and are part of the central metabolism, therefore many of these genes can be considered essential for the growth, development, and reproduction of *Mtb* (Medini et al., 2005; Tettelin et al., 2005).

In the functional analysis, the most abundant category within the genomic core after the [R] general function prediction category was the [I] Lipid transport and metabolism category. The *Mtb* cell membrane is known to be rich in lipids and approximately 40% of the dry weight of its cell wall is composed of these (Chiaradia et al., 2017). A large part of its genome encodes for lipid biosynthesis and degradation. Many of these genes are involved in the cell membrane, which allows understanding the importance not only at the structural level, but it is also known that they are implicated in functions related to cell invasion, host immune system evasion, virulence, and slow growth (Cole et al., 1998; Forrellad et al., 2013; Yang et al., 2018).

The data confirmed that the core genes are primary genes that determine the generalities of *Mtb* isolates and their lineage-4 and contain the majority of genes essential for their survival (Cole et al., 1998; Tatusov et al., 2000). Like in *Mtb*, many of the genes identified in the core of bacterial species such as *S. agalactiae*, *S. pyogenes*, and *E. coli* are housekeeping genes, most of the genes participate in basic biology aspects and are responsible for its main phenotypic characteristics (Medini et al., 2005; Vernikos et al., 2015).

### Phylogenetic analysis of the pan-genome and SNPs

Comparing the topology of the phylogenomic tree from PGM with the genotyping data generated by spoligotyping and 24-loci MIRU-VNTR, the 16 isolates previously genotyped as Haarlem1 SIT62 were part of the same clade, together with a Haarlem1 SIT3022, 14 isolates between Haarlem1 and Haarlem3 with different SIT were part of the second clade and 16 LAM isolates with different SIT made up the third clade (Figure 7). The only isolate that did not coincide with genotyping was UT277 Haarlem1 SIT3022, because it was located in the same clade of Haarlem1 SIT62. As spoligotyping and 24-loci MIRU-VNTR tests have a lower level of resolution than whole-genome analysis, the information they provide at the clade and family level is less reliable (Tatusov et al., 2000). When confirming the topology of the branches by adding 26 genomes downloaded from the NCBI belonging to the lineages (2, 3, 4, and 6), a clear grouping was observed for each lineage that was consistent with previously reported studies of the evolutionary relationships of the lineages (2, 3, and 4) considered modern and that differ from the ancient lineages (1, 5, and 6) by the deletion of the TbD1 genomic region, which in our case was observed only in the isolates belonging to the ancient lineage 6 (Supplementary Figure 6; Donoghue, 2011; Coscolla and Gagneux, 2014; Niemann and Supply, 2014). The above allowed us to infer that the topology and branch lengths obtained as a function of the genetic content (presence-absence) was adequate and can be used as a reference in future comparisons.

Single nucleotide polymorphisms are individual positions of orthologous nucleotides that vary across genomes (Leaché and Oaks, 2017). The SNPs present in the core genome were used to perform phylogenetic reconstruction of *Mtb* lineage-4 isolates. Although phylogenetic trees were reconstructed with different molecular markers for both genes and SNPs, the tree topology was preserved in the major clades and for most of the branches with each isolation (Supplementary Figure 7). The topology of the trees was consistent for the phylogenetic inference of both the pan-genome genes and the SNPs present in the genomic core of the isolates, directly correlated with the sublineages of *Mtb* lineage-4 genotyped by spoligotyping and 24 MIRU- VNTR.

Although it was expected that isolates would be grouped according to sublineage, the resolution scale for phylogenetic inference is much higher when using genes or SNPs, as compared to the DR locus and spacers analyzed from the spoligotyping technique or the tandem repeat regions present in the MIRU-VNTR that are found much less frequently in the genome. The phylogenetic trees performed with both pan-genome complete gene sequences and the concatenation of single nucleotide polymorphisms present in the genomic core were highly congruent and statistically robust. When compared with the spoligotyping and 24-loci MIRU-VNTR, a greater resolution power was observed by the genes and SNPs for being more diverse and abundant markers throughout the genome. This latter allowed us the identification of the UT277 H1 3022 isolate initially classified as a low prevalence sublineage to a high prevalence UT277 H1 SIT62 isolate.

### Pan-genome-wide association study

The pan-genome allowed the study of the association of genetic variants with the high or low prevalence trait of the isolates of *Mtb* lineage-4. In 11 genes 12 variants among insertions, deletions and SNPs were identified. Four genes with the highest statistical association with low prevalence were *mmpL12*, *PPE29*, *Rv1419*, *Rv1762c*. These encode proteins are part of the membrane or the cell wall of the mycobacterium. The remaining seven genes were significantly associated with high prevalence; *Rv3371*, *Rv2735c*, *scoA*, *mhpE*, PE-PGRS42, *lppB*, and *gabD2* which have different biological and functions in the mycobacteria.

### Low prevalence genes

We found that the *mmpL12* gene (14 of 20 isolates) had an insertion at position 1642_3000ins. This insertion of 1,358 bp generates a change in the open reading frame giving rise to two CDS of 1,740 and 1,788 bp. The insertion corresponded to a mobile genetic element IS6110, it is known that the insertion of IS6110 in coding regions of the genome generally produces an inactive gene (Gonzalo-Asensio et al., 2018). Despite having used WGS, the repetitive nature of IS6110 represented a technical challenge for both precise identification in the *mmpL12* gene as for its experimental validation, this difficulty has been reported previously to define its precise location in the chromosome due to these repetitive regions (Reyes et al., 2012; Gonzalo-Asensio et al., 2018). The presence of the IS6110 insertion in the *mmpL12* gene had already been previously identified but its effects on the gene are unknown (Reyes et al., 2012). Nevertheless, we hypothesize that the two proteins predicted as a result of the insertion in *mmpL12*, in low prevalence isolates, have most likely lost their original function, due to the loss of 5 transmembrane domains and one periplasmic domain (Supplementary Figure 8).

The proteins of the MmpL family are located in the cell membrane and their main role is the transport of lipids and siderophores directly related to the survival, virulence, and pathogenicity through the plasma membrane toward the periplasmic space (Domenech et al., 2005; Melly and Purdy, 2019). The substrate carried by this protein (MMPL12) is not yet known with certainty. However, some studies have revealed that the *mmpl12* gene is close to the *pks* and *fad* genes, which may indicate that the substrates for this protein are glyco or polyketides. An ortholog of *mmpL12* in *M. abscessus* (MAB_0855) was recently identified as a transporter for glycosyl diacylated nonadecyl diol (GDND), suggesting that the substrate for MMPL12 in *Mtb* is an undescribed cell envelope glycolipid which could exert similar functions as GDND in *M. abscessus* such as protective functions allowing survival within the host immune cells (Dubois et al., 2018).

We hypothesize that, if the mycobacteria does not have a functional MMPL12 protein that efficiently transports GDND, the cell wall will not have the basic requirements for this glycolipid to confront the host’s immune system and may be more easily cleared. This phenomenon would help to explain the low prevalence in the population of the isolates that presented insertion 1642_300ins in *mmpL12*.

The *PPE29* gene presented an insertion at position 639_641insG shifting the open reading frame in low prevalence isolates. In the wild type, the PPE29 protein is required for endothelial cell invasion and intracellular survival of *Mtb* (Gey van Pittius et al., 2006). It is likely that this insertion is affecting PPE29 protein function, reducing the ability of the bacteria to survive the action of the host immune system. As its fitness is affected, the ability to survive within cells will not be able to efficiently establish the infectious process, since it is an obligate intracellular pathogen.

The *Rv1419* gene encodes a lectin, the deletion identified at position 199delA generated a smaller protein in 14 of the 20 low prevalence genomes, which possibly inactivates its function. Lectins are a family of secretory proteins with the ability to specifically bind to carbohydrates. Several pathogens have been shown to express these molecules and to be highly involved in recognition and invasion processes (Nogueira et al., 2010). There is evidence to suggest that lectin-host interactions in *Mtb* are a potential mechanism to facilitate the establishment of infection. In this sense, it has been shown that lectins derived from *Mtb* could play an important role in infection *in vivo* (Nogueira et al., 2010; Kolbe et al., 2019). It is likely that the deletion found in the *Rv1419* gene in low prevalence isolates altered this host-pathogen interaction and therefore the ability of the mycobacterium to establish an infectious process.

Unlike the high-prevalence genomes, in 13 of the 20 low-prevalence genomes the *Rv1762c* gene contained one SNP at position 538C > T, producing a premature stop codon. Sequence analysis suggests it encodes for a non-essential protein that has two putative heavy metal-binding domains, located in a fraction of the cell membrane. It is likely to be related to the regulation or transport of metal ion concentrations across the membrane as a physiological mechanism between the mycobacteria-host (Supplementary Figure 9; Agranoff and Krishna, 2004).

### High prevalence genes

We found that the *Rv3371* gene had a SNP at position 374T > A and a deletion at position 376delA in 16 of the 27 high-prevalence genomes, which generate an open reading frame shift and result in a smaller protein that is probably not functional. These results are consistent with previous studies, as this deletion was found only in high-prevalence isolates (Daniel et al., 2004; Sirakova, 2006). The *Rv3371* gene encodes for the enzyme diacylglycerol acyltransferase, involved in the synthesis of triacylglycerols, these are important in the survival of the bacterium when it is in a latent state. The mycobacteria store lipids in intracellular inclusion bodies in the form of triacylglycerols which will be a source of carbon and fatty acids during the latent state. The *Rv3371* promoter has been shown to be under-expressed when mycobacteria are in a state of active replication *in vitro* and is overexpressed when the bacilli enter conditions of hypoxia and low metabolic activity (Daniel et al., 2004; Sirakova, 2006). Studies with mutants of the *Rv3371* gene found that mycobacteria could not enter the non-replicative phase under conditions of hypoxia, nitrosative stress, or iron depletion; that is, they did not have the ability to go into latency. This inability was due to the low formation of vesicles that stored triacylglycerols. The deletion of this gene did not affect the *in vitro* growth of the mycobacteria or the morphology of the colonies (Daniel et al., 2004; Sirakova, 2006). It is likely that these isolates present an inability to enter a state of latency and this contributes to more cases of active TB, associated with an increase in the transmission of these isolates.

The *scoA* gene was found over-expressed in a study carried out with a persistence model, which indicates that this gene is probably involved in the metabolism of mycobacteria which are in a state of latency (Fleischmann et al., 2002). In this work, the scoA gene showed the 476insG insertion, which generated a change in the codon (TAT) for a stop codon (TAG) at position 159, truncating the protein. The effect of this mutation is most likely inactivation of the protein. This gene encodes the enzyme succinyl-CoA:3-ketoacid-coenzyme transferase subunit A, which is used by mycobacteria for the utilization of ketones resulting from B-oxidation. Studies in mycobacteria suggest that dormant bacilli use fatty acids as their predominant source of energy through β-oxidation pathways (Fleischmann et al., 2002). It is possible that this insertion in *scoA* is affecting the ability to go into the state of latency of the mycobacteria and this favors it to remain in a state of active replication, facilitating its transmission and high prevalence.

The *Rv2735c* gene encodes a hypothetical conserved 330 amino acid protein with unknown function, 16 of the 27 high prevalence isolates present an insert at position 363_364insGT displacing the open reading frame. This generates a truncated protein of 159 amino acids, which is possibly not functional. The *lppB* gene encodes a lipoprotein which has not yet have a known function (Sutcliffe and Harrington, 2004). Lipoproteins are found in the cell envelope and help support this structure. Several of them have been characterized as virulence factors (Sutcliffe and Harrington, 2004). In this work, this gene presented a SNP at position 362C > A, this polymorphism produces a non-synonymous mutation changing the codon (TCG) of a serine (S) for a stop codon (TAG), truncating the protein at position 120 of the open reading frame. It is difficult to hypothesize why these genes are possibly truncated in high prevalence isolates. Functional studies would be needed to determine their role and the possible effect in high prevalence isolates.

The *mhpE* and *gabD2* genes encode the enzymes 4-hydroxy-2-oxopentanoic acid aldolase and succinate-semialdehyde dehydrogenase, respectively. These enzymes are involved with intermediates of the tricarboxylic acid cycle such as pyruvate (*mhpE*) and succinate (*gabD2*) (Tian et al., 2005; Carere et al., 2013). When mycobacteria are found under hypoxic or intracellular conditions within macrophages, these enzymes participate in alternative pathways to the tricarboxylic acid cycle for obtaining energy (Tian et al., 2005; Carere et al., 2013). These alternate pathways appear to be most active when the bacteria enter in a reduction of metabolic activity. In this work, the *mhpE* and *gabD2* genes presented a SNP and insertion, respectively. These changes were observed in the high prevalence isolates, which could be indicating that these isolates, being in a phase of active replication and circulating within a population, it may not be necessary to activate alternative routes of the tricarboxylic acid cycle.

The genetic variants identified in this association study to the prevalence were part of the *Mtb* dispensable genome. The importance of these gene variants in transmission, pathogenicity and virulence of *Mtb* has not yet been experimentally proven. Experimental trials in animal models infected with high and low prevalence *Mtb* isolates, in addition to the use of CRISPR technology for editing the genes identified with association to prevalence, should be carried out to demonstrate their role in pathogenicity and transmission. Similar trials of infection and transmission of *Mtb* have been carried out in mini pigs, proving to be a suitable model for the study of infection and natural transmission of TB (Clark et al., 2014; Choudhary et al., 2015; Ramos et al., 2017).

Despite the fact that the pan-genome association study was performed independent of the sublineage, the phylogenomic analysis together with the pan-GWAS showed that most of the differential variants identified in the high or low prevalence of *Mtb* also showed an association with the sublineage. When comparing the topology of the *Mtb* isolates in the clades of the trees grouped by their close evolutionary relationships with the differential variants between high and low prevalence, it was observed that the presence or absence of the variant was related to certain sublineages. Suggesting that particular dominant sublineages such as LAM9 SIT42 and H1 SIT62 have evolutionary advantages that could be due to the genetic variants associated with the high prevalence of these sublineages over the other sublineages present in the NE area of Medellín. The above allows us to infer that the high prevalence trait is not given by a single polymorphism or variant, but by multiple variants in the genome that probably contribute to the success of *Mtb* sublineages LAM9 SIT42 and H1 SIT62.

Several studies have shown that lineages and sublineages are associated with virulence and transmission traits. For example, the outbreak of the most prevalent *Mtb* lineage in Scandinavia, the specific strain 2/1112-15 (C2), between the years 1992 to 2014 in Denmark the rapid transmission of the C2 genotype was evidenced, suggesting that specific virulence factors added poor TB control favored the success of this genotype (Folkvardsen et al., 2018). In another study, the association of the LAM RD^Rio^ sublineage with drug resistance has been demonstrated; 33% of a total of 857 *Mtb* isolates analyzed in Portugal were due to the strain RD^Rio^ and represented more than 60% of the MDR strains, the most predominant sublineage was the RD^Rio^ LAM1 SIT20. Analysis with 12 loci MIRU-VNTR of the RD^Rio^ strain revealed that 96.3% (129/134) of the MDR and XDR clusters belonged to RD^Rio^ strains (David et al., 2012). Strains of the Beijing lineage demonstrated an increase in their fitness of transmission when they were resistant to streptomycin when compared with strains of the EAI lineage. Inferring that streptomycin resistance, contrary to popular belief, gives Beijing strains an advantage in fitness compared to other genotypes (Buu et al., 2012).

It is likely that the adaptive advantages of certain lineages or sublineages improve their fitness, generating compensatory mutations allowing them to be more successful. In contrast, in *Mtb* lineages with low prevalence their fitness cost is higher, making the process of transmission and infection difficult in a new host. Variants have been identified in genes under positive selection, with association to the virulence of *Mtb*, these have shown the expansion of clones (Beijing) in different branches, making them more successful than others (Merker et al., 2015). Strains of this lineage have been suggested to have selective advantages compared to other strains of MTBC lineages, such as increasing their ability to acquire drug resistance, linked to the high frequency of mutations, many of them compensatory mutations that mitigate the fitness cost created by resistance mutations. This increases their transmissibility, virulence, and favors a rapid progression to disease after infection (Caminero et al., 2001; Jou et al., 2005; Cowley et al., 2008; Buu et al., 2012). This heterogeneity suggests the existence of sublineages with biogeographic diversity, which present particular pathogenic properties associated with their biology (Caminero et al., 2001; Jou et al., 2005; Cowley et al., 2008; Buu et al., 2012).

Our results suggest that the variants identified in the LAM9 SIT42 and H1 SIT62 high prevalence sublineages possibly contribute to their virulence, pathogenicity, or the ability to transmission in a community, conferring genetic advantages that favor the transmission success of these isolates compared to the less prevalent isolates. In these latter sublineages, the variants that are affecting the functionality of the *mmpL12*, *PPE29*, *Rv1419*, and *Rv1762c* genes may have a fitness cost, which is reflected in a possible decrease in their ability to generate an active infection. Interestingly, the *mmpL12*, *PPE29*, *Rv1419* genes are found related to intracellular recognition, invasion, and survival processes directly involved in host-pathogen interaction (Domenech et al., 2005; Gey van Pittius et al., 2006; Nogueira et al., 2010). It is possible that the polymorphisms found in these genes have a high fitness cost in the low prevalence isolates, making their transmission difficult. It is necessary to continue increasing the number of isolates in this type of study to determine the generality of these variants in different lineages and sublineages that behave with phenotypic traits of high and low prevalence in other geographic areas of Colombia and the world and thus verify the universality of the results.

## Data availability statement

The data presented in this study are deposited in the https://www.ncbi.nlm.nih.gov/ repository, accession number: PRJNA838941.

## Author contributions

JR, FR, UH-P, and RA contributed to the conception and design of the study. NÁ performed the DNA isolation and PCR validations. UH-P and BC-M carried out the bioinformatic and statistical analysis. UH-P wrote the first draft of the manuscript. All authors contributed to the manuscript revision, read, and approved the submitted version.
