# Supplementary figures and images for: Pan-genome association study of Mycobacterium tuberculosis lineage-4 revealed specific genes related to the high and low prevalence of the disease in patients from the North-Eastern area of Medellín, Colombia
Source: Front Microbiol. 2023 Jan 4;13:1076797. doi: 10.3389/fmicb.2022.1076797 (PMC9846648; doi:10.3389/fmicb.2022.1076797)

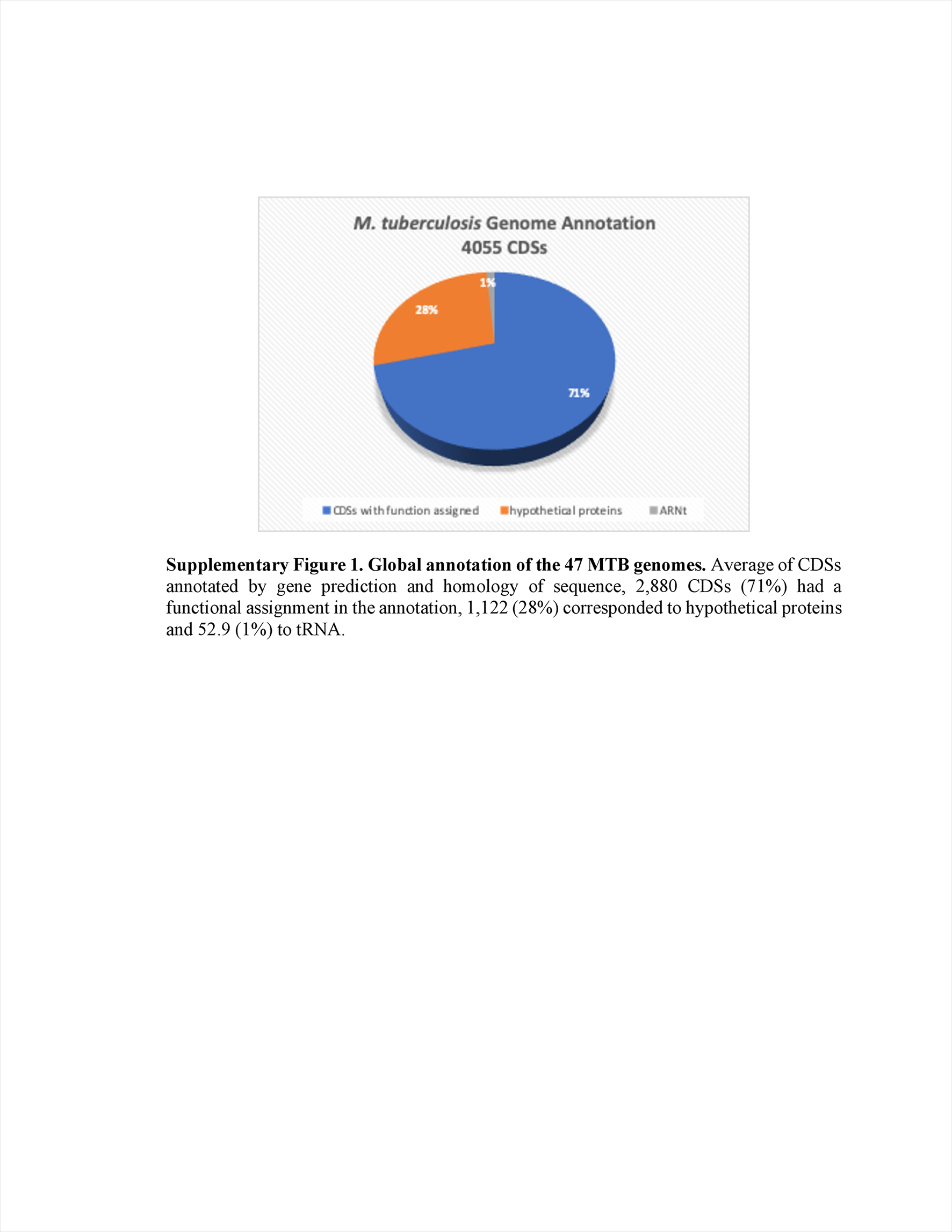

Supplement: Supplementary file 1 [file Image_1.TIFF]

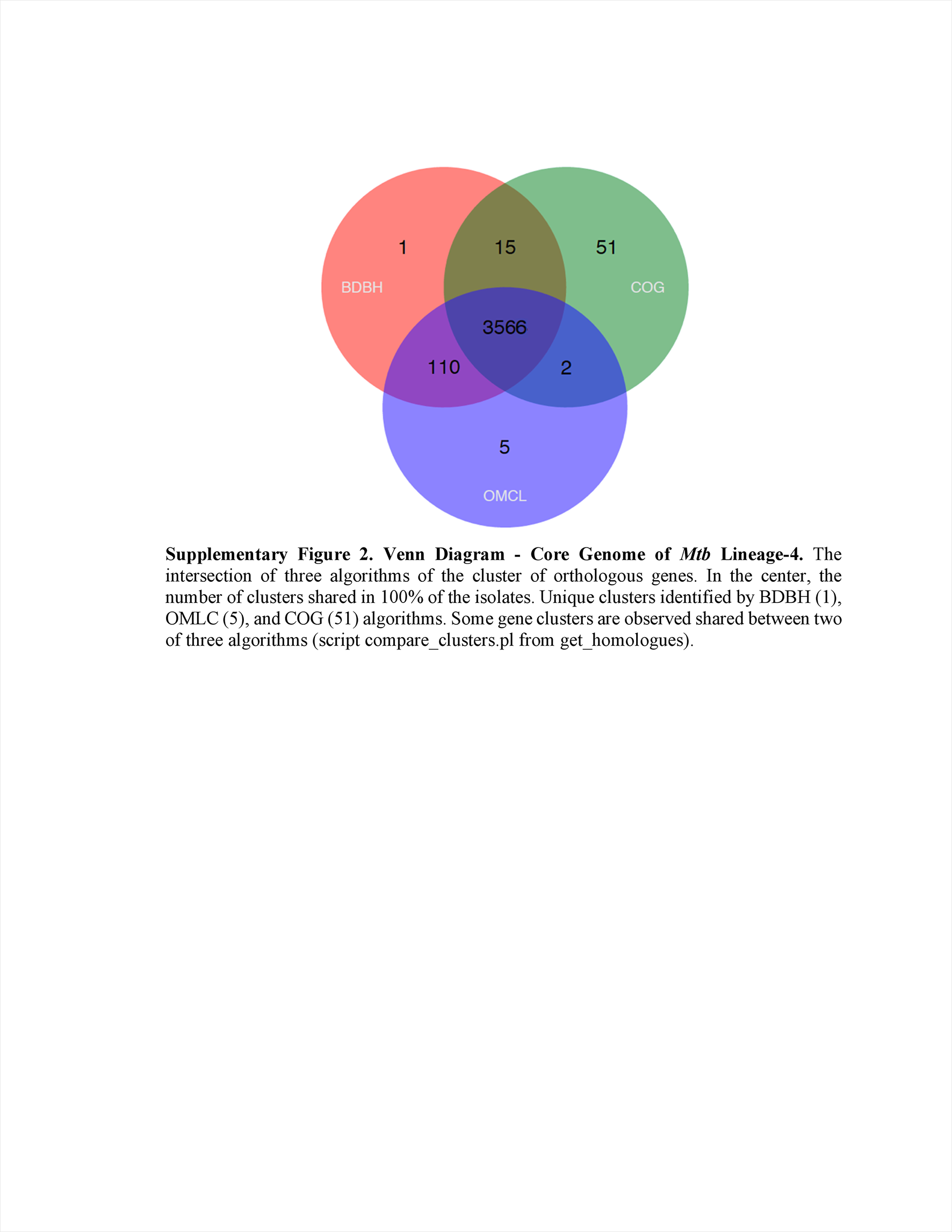

Supplement: Supplementary file 2 [file Image_2.TIFF]

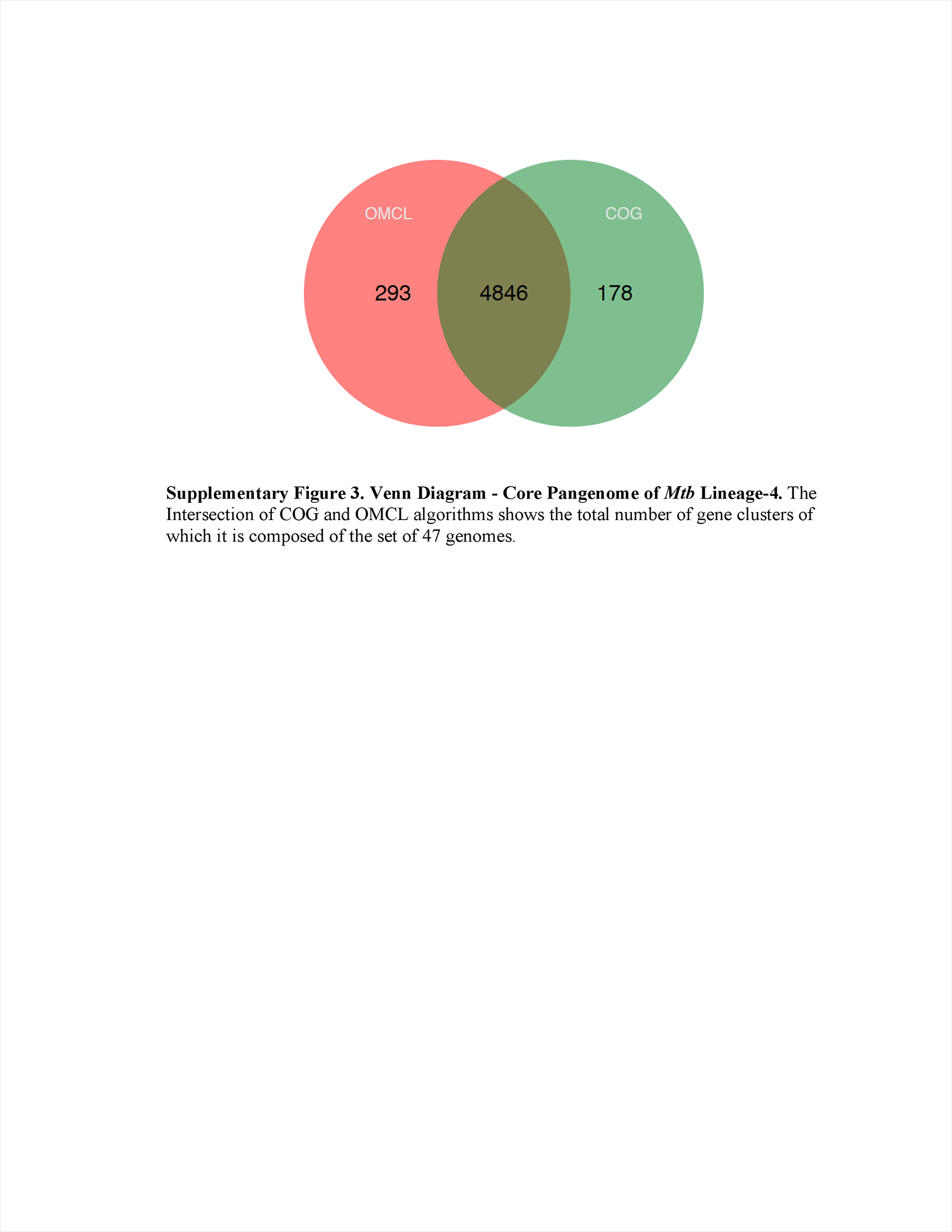

Supplement: Supplementary file 3 [file Image_3.TIFF]

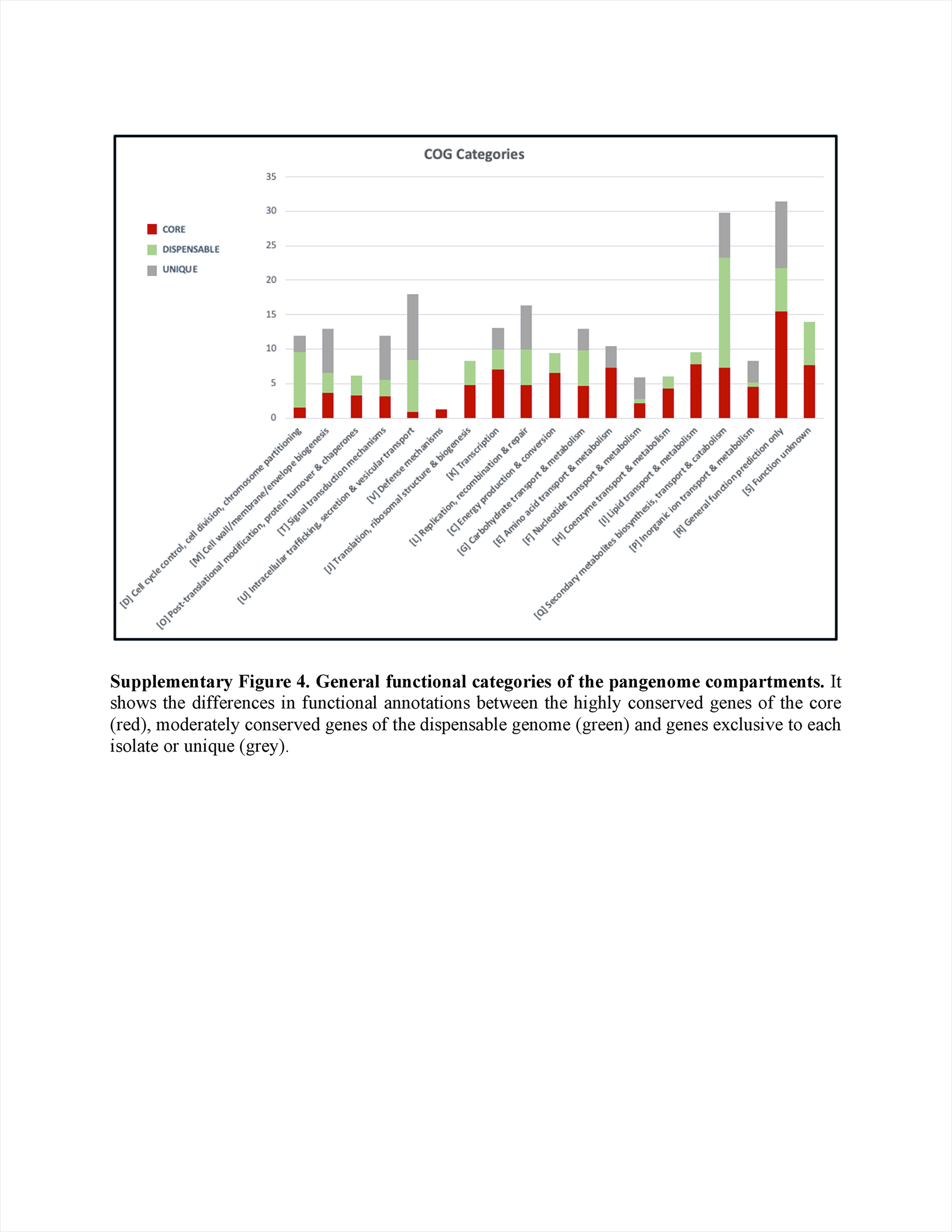

Supplement: Supplementary file 4 [file Image_4.tiff]

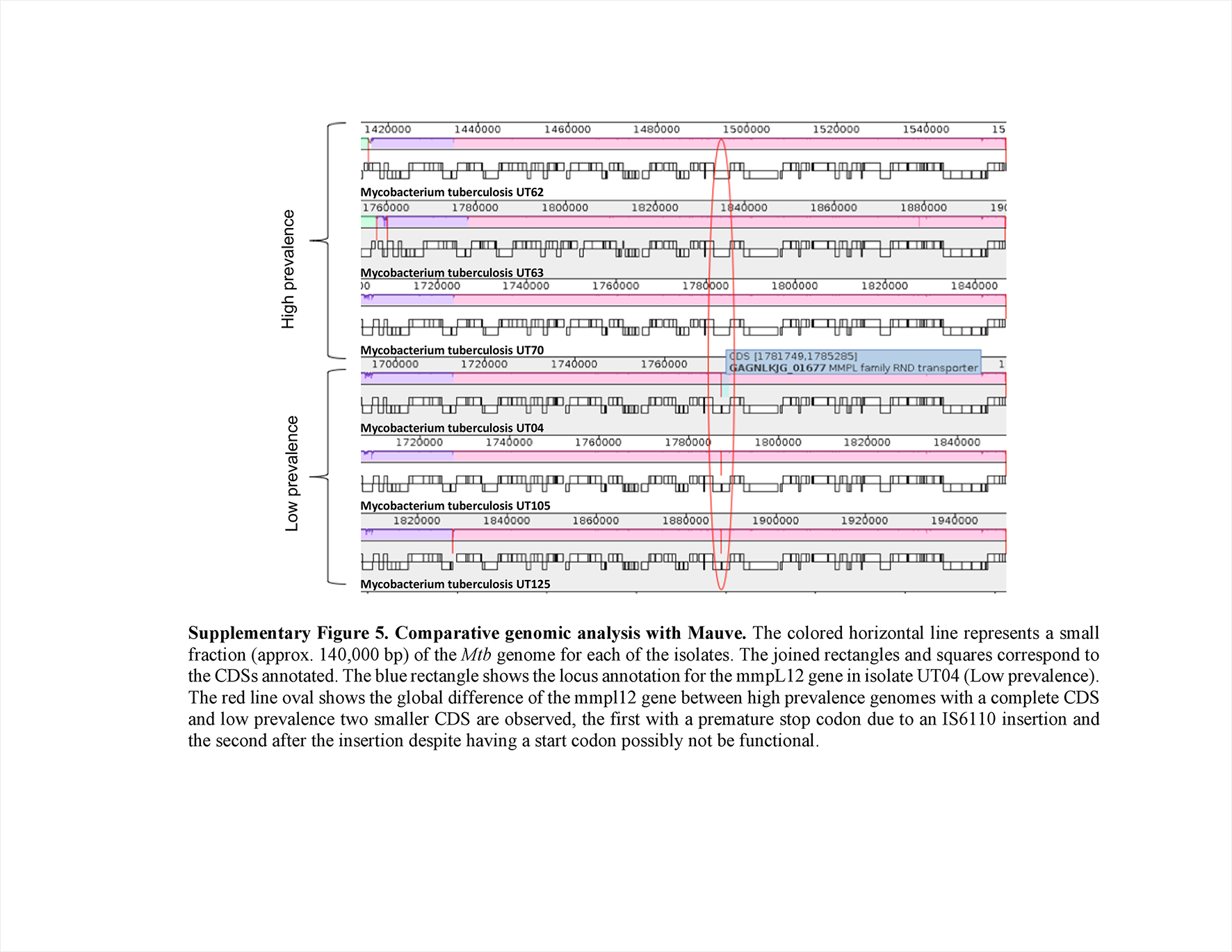

Supplement: Supplementary file 5 [file Image_5.tiff]

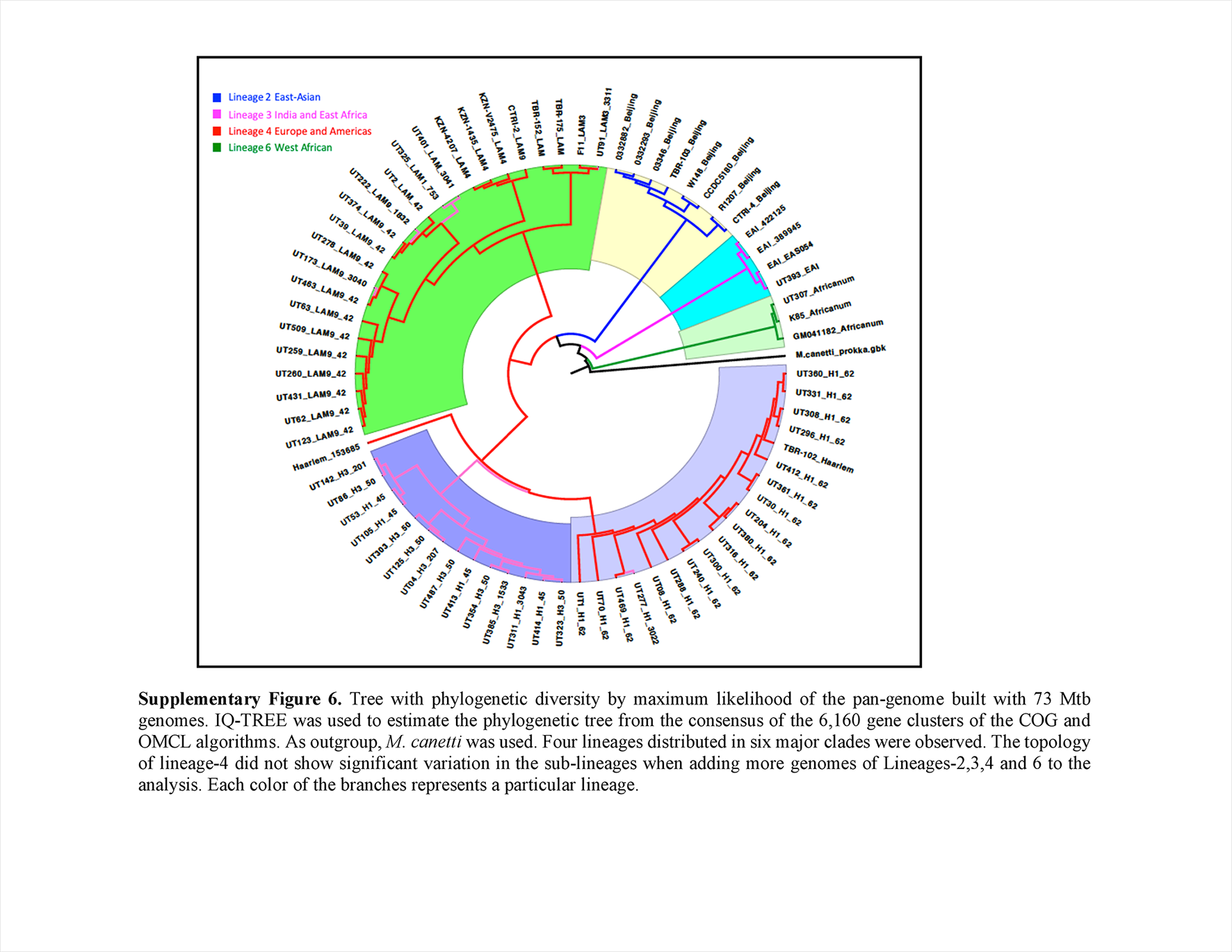

Supplement: Supplementary file 6 [file Image_6.tiff]

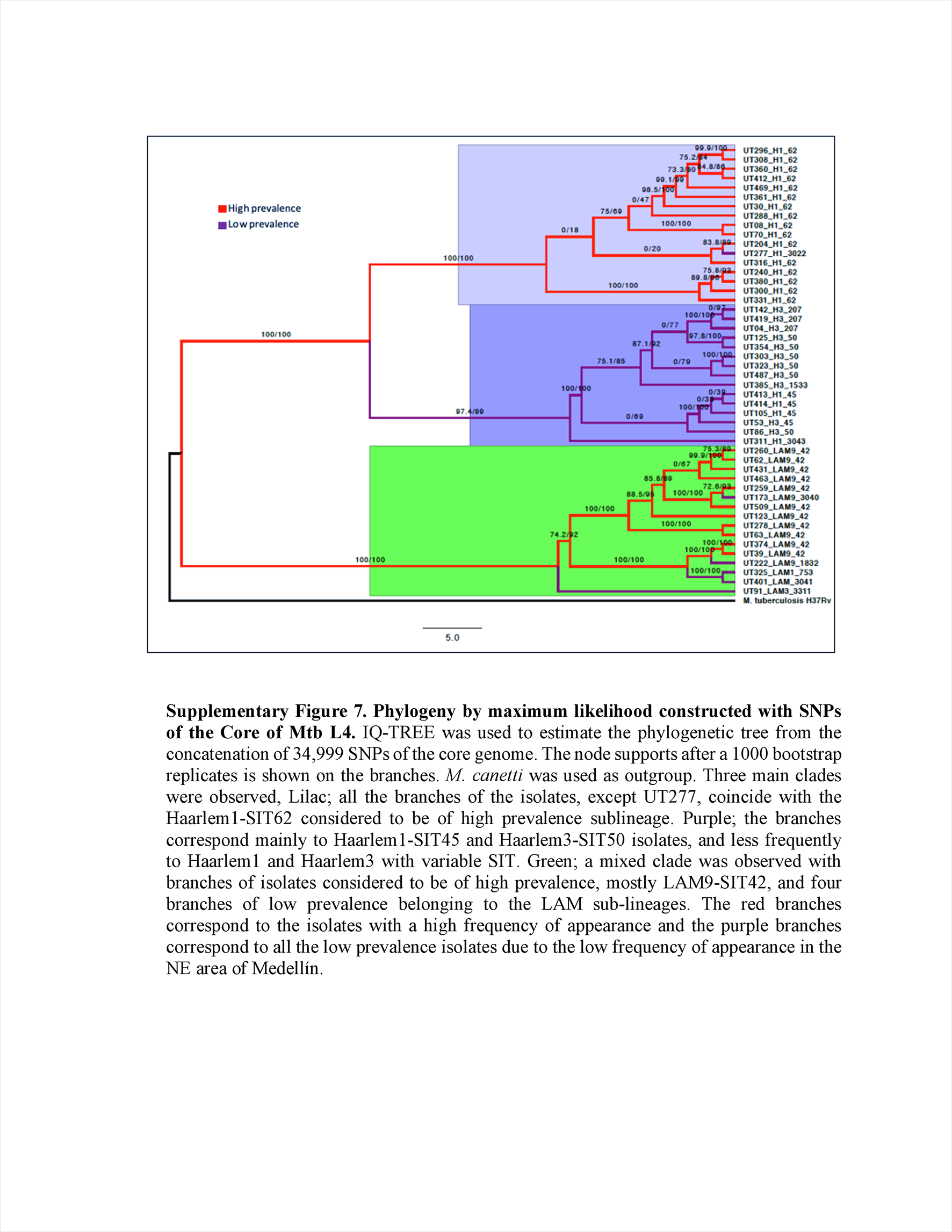

Supplement: Supplementary file 7 [file Image_7.tiff]

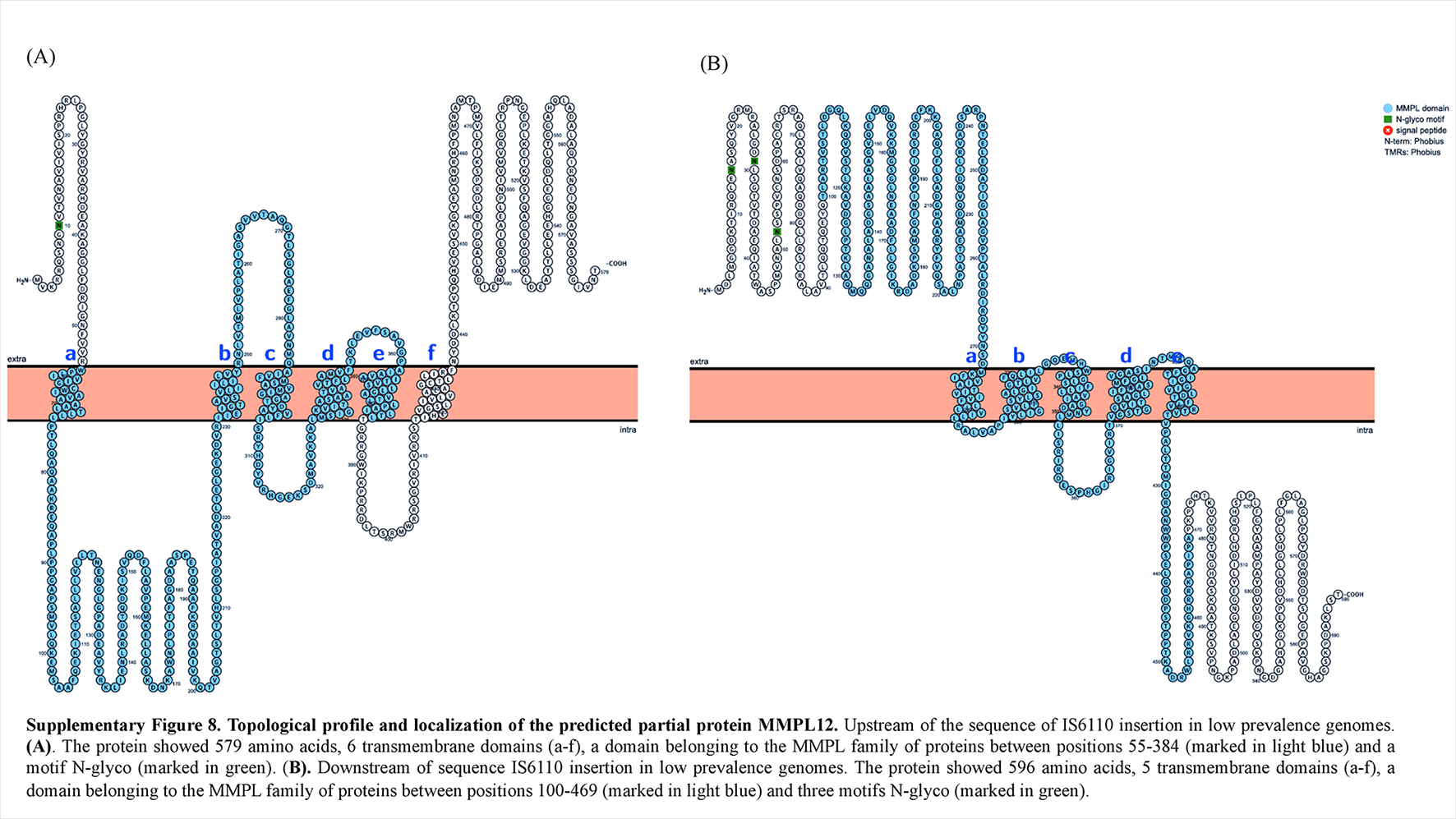

Supplement: Supplementary file 8 [file Image_8.tiff]

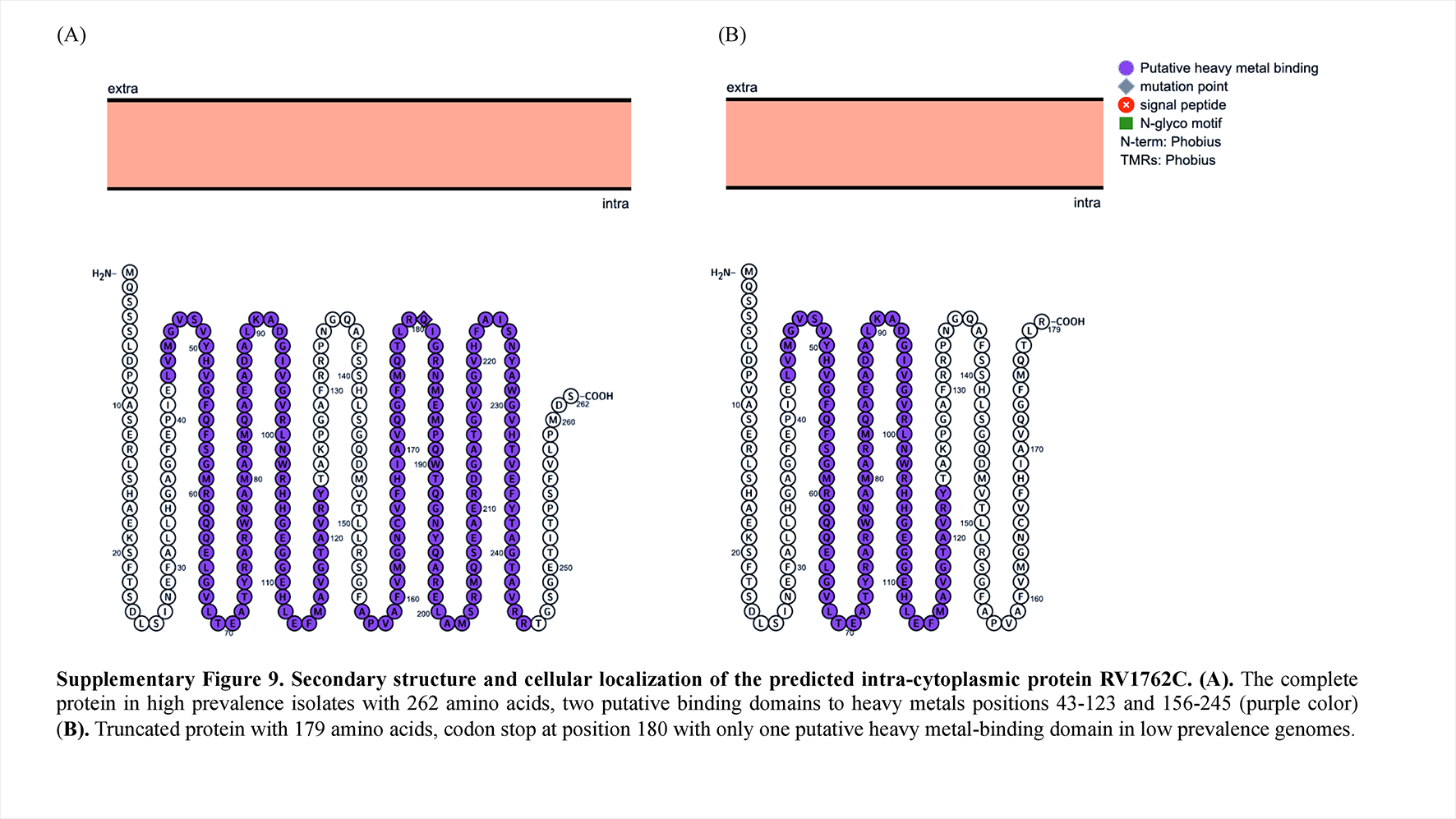

Supplement: Supplementary file 9 [file Image_9.tiff]
