# Supplementary material for: Pan-genome association study of Mycobacterium tuberculosis lineage-4 revealed specific genes related to the high and low prevalence of the disease in patients from the North-Eastern area of Medellín, Colombia
Source: Front Microbiol. 2023 Jan 4;13:1076797. doi: 10.3389/fmicb.2022.1076797 (PMC9846648; doi:10.3389/fmicb.2022.1076797)
Supplement: Supplementary file 12 [file Data_Sheet_3.PDF]

Supplementary Table 3. General features of 47 *Mtb* genomes belonging lineage-4. Clinical isolates with their prevalence characteristic described in material and methods. Statistical metrics for each genome *de novo* assembled using Quast. Contigs[a] >=500, represent the lower threshold in base pairs for the contig length that was considered.

Genome Number	Isolate name	Prevalence	Coverage (X)[a]	N50 (pb)	Largest contig (pb)	No. of contigs	Total length (Mb)
1	UT04	Low	120	144,181	243,874	88	4.364
2	UT105	Low	172	166,712	301,515	66	4.364
3	UT125	Low	114	128,377	301,515	91	4.360
4	UT142	Low	195	164,374	301,458	74	4.367
5	UT173	Low	116	163,378	255,747	84	4.354
6	UT222	Low	180	143,119	255,730	82	4.316
7	UT277	Low	123	125,587	257,580	96	4.372
8	UT303	Low	158	125,587	258,576	89	4.355
9	UT311	Low	74	81,916	233,135	119	4.338
10	UT323	Low	37	79,518	228,280	127	4.323
11	UT325	Low	85	142,712	260,503	112	4.326
12	UT354	Low	51	90,432	257,703	122	4.352
13	UT385	Low	112	83,179	228,198	125	4.361
14	UT401	Low	53	114,802	261,188	131	4.31
15	UT412	High	43	81,782	228,214	124	4.346
16	UT413	Low	143	114,927	259,098	102	4.351
17	UT414	Low	50	78,270	228270	127	4.329
18	UT469	High	189	123,215	302,814	85	4.372
19	UT487	Low	129	81,566	196,627	140	4.336
20	UT509	High	120	126,470	255,846	116	4.340
21	UT53	Low	179	166,765	301,686	70	4.363
22	UT86	Low	198	166,879	301,797	69	4.377
23	UT91	Low	156	161,924	301,518	75	4.366
24	UT08	High	136	123,237	303,014	101	4.365
25	UT123	High	50	90,407	341,045	135	4.339
26	UT204	High	37	81,319	228,227	139	4.347
27	UT240	High	62	93,519	228,230	100	4.341
28	UT259	High	119	128,374	256,079	111	4.339
29	UT260	High	77	129,940	256,900	88	4.345
30	UT278	High	153	163,403	255,484	67	4.353
31	UT288	High	69	98,814	230,665	95	4.360
32	UT296	High	136	115,584	302,683	88	4.364
33	UT300	High	58	89,088	257,668	115	4.354
34	UT308	High	31	82,891	228,156	102	4.346
35	UT30	High	60	81,738	258,558	129	4.342
36	UT316	High	42	83,037	228,228	132	4.354
37	UT331	High	30	83,006	261,380	127	4.341
38	UT360	High	33	82,669	231,206	102	4.345
39	UT361	High	63	83,885	228,170	122	4.336
40	UT374	High	126	123,106	261,164	109	4.312
41	UT380	High	84	83,009	342,907	125	4.341
42	UT39	High	122	129,796	261,164	95	4.319
43	UT431	High	66	116,180	248,296	117	4.339
44	UT463	High	132	117,544	255,735	105	4.357
45	UT62	High	52	97,882	215,851	122	4.340
46	UT63	High	77	133,611	255,420	112	4.340
47	UT70	High	189	130,474	301,924	79	4.374
