# Supplementary material for: Pan-genome association study of Mycobacterium tuberculosis lineage-4 revealed specific genes related to the high and low prevalence of the disease in patients from the North-Eastern area of Medellín, Colombia
Source: Front Microbiol. 2023 Jan 4;13:1076797. doi: 10.3389/fmicb.2022.1076797 (PMC9846648; doi:10.3389/fmicb.2022.1076797)
Supplement: Supplementary file 13 [file Data_Sheet_4.PDF]

Supplementary Table 4. Error correction by reads mapping to *de novo* assembly using Pilon. Representative example for UT417 and UT30: Each genome presented some type of error or assembly discrepancies ranging from a single incorrect base in a single contig to small sequence segments no greater than 5 consecutive nucleotides not concordant in one or more contigs, as well as small gaps that could not be resolved.

Isolate	Contig	Position	Identified error	Correction
UT487	1	107441	A	G
	15	40610-40619	NNNNNNNNNN	TTTTGA
	35	4510	C	A
	35	6588	.	A
	35	6589-6598	NNNNNNNNNN	ACA
	23	76192	C	T
	23	76196	G	C
	100	554	G	T
	64	262	G	C
	64	272	C	T
	64	572	A	C
	64	601	C	A
	85	623-649	CAGATAACGCAACGCCGCAGGCGCGCG	TAGATAACGCAACGCCGCAGGCGCGCA
UT30	99	716-741	GGCAAGGATCTGCGCAATGCGGTCCG	CAGCATGTACTGATGCATCCGGGGAC
	35	174	C	G
	38	76	T	C
