# Supplementary material for: Pan-genome association study of Mycobacterium tuberculosis lineage-4 revealed specific genes related to the high and low prevalence of the disease in patients from the North-Eastern area of Medellín, Colombia
Source: Front Microbiol. 2023 Jan 4;13:1076797. doi: 10.3389/fmicb.2022.1076797 (PMC9846648; doi:10.3389/fmicb.2022.1076797)
Supplement: Supplementary file 14 [file Data_Sheet_5.PDF]

Supplementary Table 5. Lists of high or low prevalence isolates that presented association with the genes identified.

Gene	High prevalence (n27)			Low prevalence (n14)		
	Isolate	Sublineaje	SIT	Isolate	Sublineaje	SIT
mmpL12 PPE29 Rv1419	UT08	H1	62	UT04	H3	207
	UT123	LAM9	42	UT105	H1	45
	UT204	H1	62	UT125	H3	50
	UT240	H1	62	UT142	H3	201
	UT259	LAM9	42	UT303	H3	50
	UT260	LAM9	42	UT311	H1	3043
	UT278	LAM9	42	UT323	H3	50
	UT288	H1	62	UT354	H3	50
	UT296	H1	62	UT385	H3	1533
	UT300	H1	62	UT413	H1	45
	UT308	H1	62	UT414	H1	45
	UT30	H1	62	UT487	H3	50
	UT316	H1	62	UT53	H1	45
	UT331	H1	62	UT86	H3	50
	UT360	H1	62			
	UT361	H1	62			
	UT374	LAM9	42			
	UT380	H1	62			
	UT39	LAM9	42			
	UT412	H1	62			
	UT431	LAM9	42			
	UT463	LAM9	42			
	UT469	H1	62			
	UT509	LAM9	42			
	UT62	LAM9	42			
	UT63	LAM9	42			
	UT70	H1	62			

Gene	High prevalence (n25)			Low prevalence (n13)		
	Isolate	Sublineaje	SIT	Isolate	Sublineaje	SIT
Rv1762c	UT08	H1	62	UT04	H3	207
	UT123	LAM9	42	UT105	H1	45
	UT204	H1	62	UT125	H3	50
	UT240	H1	62	UT142	H3	201
	UT259	LAM9	42	UT303	H3	50
	UT260	LAM9	42	UT323	H3	50
	UT278	LAM9	42	UT354	H3	50
	UT288	H1	62	UT385	H3	1533
	UT296	H1	62	UT413	H1	45
	UT300	H1	62	UT414	H1	45
	UT308	H1	62	UT487	H3	50
	UT30	H1	62	UT53	H1	45
	UT316	H1	62	UT86	H3	50
	UT331	H1	62			
	UT360	H1	62			
	UT361	H1	62			
	UT380	H1	62			
	UT412	H1	62			
	UT431	LAM9	42			
	UT463	LAM9	42			
	UT469	H1	62			
	UT509	LAM9	42			
	UT62	LAM9	42			
	UT63	LAM9	42			
	UT70	H1	62			

Gene	High prevalence (n16)			Low prevalence (n19)		
	Isolate	Sublineaje	SIT	Isolate	Sublineaje	SIT
Rv3371 Rv2735c scoA mhpE lppB gabD2	UT08	H1	62	UT04	H3	207
	UT204	H1	62	UT105	H1	45
	UT240	H1	62	UT125	H3	50
	UT288	H1	62	UT142	H3	201
	UT296	H1	62	UT173	LAM9	3040
	UT300	H1	62	UT222	LAM9	1832
	UT308	H1	62	UT303	H3	50
	UT30	H1	62	UT311	H1	3043
	UT316	H1	62	UT323	H3	50
	UT331	H1	62	UT325	LAM1	753
	UT360	H1	62	UT354	H3	50
	UT361	H1	62	UT385	H3	1533
	UT380	H1	62	UT401	LAM	3041
	UT412	H1	62	UT413	H1	45
	UT469	H1	62	UT414	H1	45
	UT70	H1	62	UT487	H3	50
				UT53	H1	45
				UT86	H3	50
				UT91	LAM3	3311

Gene	High prevalence (n16)			Low prevalence (n16)		
	Isolate	Sublineaje	SIT	Isolate	Sublineaje	SIT
PE-PGRS42	UT08	H1	62	UT04	H3	207
	UT204	H1	62	UT105	H1	45
	UT240	H1	62	UT125	H3	50
	UT288	H1	62	UT142	H3	201
	UT296	H1	62	UT173	LAM9	3040
	UT300	H1	62	UT303	H3	50
	UT308	H1	62	UT311	H1	3043
	UT30	H1	62	UT323	H3	50
	UT316	H1	62	UT325	LAM1	753
	UT331	H1	62	UT354	H3	50
	UT360	H1	62	UT385	H3	1533
	UT361	H1	62	UT401	LAM	3041
	UT380	H1	62	UT413	H1	45
	UT412	H1	62	UT487	H3	50
	UT469	H1	62	UT53	H1	45
	UT70	H1	62	UT86	H3	50
