# Supplementary material for: Pan-genome association study of Mycobacterium tuberculosis lineage-4 revealed specific genes related to the high and low prevalence of the disease in patients from the North-Eastern area of Medellín, Colombia
Source: Front Microbiol. 2023 Jan 4;13:1076797. doi: 10.3389/fmicb.2022.1076797 (PMC9846648; doi:10.3389/fmicb.2022.1076797)
Supplement: Supplementary file 15 [file Data_Sheet_6.PDF]

Supplementary Table 6. Variants identified in genes associated with high or low prevalence. The pairs of primers that amplified the region of interest are observed and their PCR products were selected for Sanger sequencing.

Gene	Variant	PCR product (pb) High prevalence	PCR product (pb) Low prevalence	Primer forward 5´-3´	Tm ºC	%GC	Primer Reverse 5´-3´	Tm ºC	%GC
mmpL12	1642_3000ins	350	1650	CAAGGTCTCGTTTCAGGCTG	63	55	GTTGAGGGCGTTGACCATAG	64	55
PPE29	639_641insG	332	331	ATGACAGCGTTCACTGAGCC	64.3	55	CTAACAGGTATGGCACCCCG	64.2	60
Rv1419	199delA	341	340	GATTGGGCGATGTTTGCCTG	63.7	55	ACGCTATCCCACTGTTGGTC	63.7	55
Rv1762c	538C>T	357	357	GGGAACACCTTGAGTTCATGG	64.52	52.38	GCGTAGTTCGAGATGGCGA	63.1	58
Rv3371	374T>A, 376delA	343	344	CGACTATCCGGGATTCGACC	63.9	60	GACATTCGCCACAGCGTTTC	63.9	55
Rv2735c	363_364insGT	280	278	TTTCCCGGTGACGGTAGGTA	64.6	55	CGGGTGACCTGCTCGATAAA	63.8	55
scoA	476_478insG	310	309	GGGCATACCGGCCTTCTATAC	63.7	57.1	TCACCCGGCTCGACCAAGT	63.7	60
mhpE	169G>T	332	332	CAGAGAGCCGATTGTCCTGG	64	60	AATTCGGTGGCGATGACACA	64.3	50
PE-PGRS42	1382_1417del	300	336	GGTGGTATCGGTGGTGACG	65	63	CCACCATCACCACCTACGC	65	60
lppB	362C>A	300	300	GACAAATCACCGCACCTGAC	64	55	TGGCGGCAATCTTGAAGTCT	63.7	50
gabD2	610_644ins	532	498	GATCTCGCCGTGGAACTACC	64	60	GTGCCGAGCTTCATGTTCCG	63.4	50
