# Supplementary material for: Pan-genome association study of Mycobacterium tuberculosis lineage-4 revealed specific genes related to the high and low prevalence of the disease in patients from the North-Eastern area of Medellín, Colombia
Source: Front Microbiol. 2023 Jan 4;13:1076797. doi: 10.3389/fmicb.2022.1076797 (PMC9846648; doi:10.3389/fmicb.2022.1076797)
Supplement: Supplementary file 10 [file Data_Sheet_1.PDF]

Supplementary Table 1. Genotyping of High prevalence Mtb Clinical isolates in the northeastern area of Medellín. The spoligotyping shows patterns of presence (black square) or absence (white square) of some of the 43 spacers of the DR locus, as well as the sub-lineage and the number of Spoligo-International-Type (SIT). Each position of the 24-loci MIRU-VNTR represents the number Interspersed Repetitive Units-Variable-Number of Tandem Repeats of a particular locus, useful for discrimination between isolates and the MIRU code to know its geographical location.

Isolate	Year	Spologotype pattern	Sub-lineage	SIT	24 MIRU-VNTR loci	24-MIT
UT08	2005	■■■■■■■■■■■■■■■■■■■■■■■■■□□□□□□■□□□□■■■□■■■	H1	62	225325153323333574234423	183
UT123	2005	■■■■■■■■■■■■■■■■■■■■□□□□■■■■■■■■□□□□■■■■■■■	LAM9	42	124325153220224262334132	o
UT204	2005	■■■■■■■■■■■■■■■■■■■■■■■■■□□□□□□■□□□□■■■□■■■	H1	62	225325153323333574234423	183
UT240	2005	■■■■■■■■■■■■■■■■■■■■■■■■■□□□□□□■□□□□■■■□■■■	H1	62	225225153323333574234423	201
UT259	2006	■■■■■■■■■■■■■■■■■■■■□□□□■■■■■■■■□□□□■■■■■■■	LAM9	42	125326153220224262334132	185
UT260	2006	■■■■■■■■■■■■■■■■■■■■□□□□■■■■■■■■□□□□■■■■■■■	LAM9	42	124326153220224262334132	187
UT278	2006	■■■■■■■■■■■■■■■■■■■■□□□□■■■■■■■■□□□□■■■■■■■	LAM9	42	124326153220224262334122	218
UT288	2006	■■■■■■■■■■■■■■■■■■■■■■■■■□□□□□□■□□□□■■■□■■■	H1	62	225325154325333574234423	o
UT296	2006	■■■■■■■■■■■■■■■■■■■■■■■■■□□□□□□■□□□□■■■□■■■	H1	62	225325153323233464234424	o
UT30	2005	■■■■■■■■■■■■■■■■■■■■■■■■■□□□□□□■□□□□■■■□■■■	H1	62	225325153323233474334424	190
UT300	2006	■■■■■■■■■■■■■■■■■■■■■■■■■□□□□□□■□□□□■■■□■■■	H1	62	226325154323333574234423	210
UT308	2006	■■■■■■■■■■■■■■■■■■■■■■■■■□□□□□□■□□□□■■■□■■■	H1	62	225325133324233474234424	o
UT316	2006	■■■■■■■■■■■■■■■■■■■■■■■■■□□□□□□■□□□□■■■□■■■	H1	62	225325153323323574234425	o
UT331	2006	■■■■■■■■■■■■■■■■■■■■■■■■■□□□□□□■□□□□■■■□■■■	H1	62	226325154323333574234423	210
UT360	2006	■■■■■■■■■■■■■■■■■■■■■■■■■□□□□□□■□□□□■■■□■■■	H1	62	225325153423233474234424	223
UT361	2006	■■■■■■■■■■■■■■■■■■■■■■■■■□□□□□□■□□□□■■■□■■■	H1	62	225325123323233464234424	o
UT374	2006	■■■■■■■■■■■■■■■■■■■■□□□□■■■■■■■■□□□□■■■■■■■	LAM9	42	222126132321214132234152	o
UT380	2006	■■■■■■■■■■■■■■■■■■■■■■■■■□□□□□□■□□□□■■■□■■■	H1	62	225325153323333574234423	183
UT39	2005	■■■■■■■■■■■■■■■■■■■■□□□□■■■■■■■■□□□□■■■■■■■	LAM9	42	222126132321214132434152	192
UT412	2006	■■■■■■■■■■■■■■■■■■■■■■■■■□□□□□□■□□□□■■■□■■■	H1	62	226325153323233474234424	220
UT431	2006	■■■■■■■■■■■■■■■■■■■■□□□□■■■■■■■■□□□□■■■■■■■	LAM9	42	124326153220224262334132	187
UT463	2006	■■■■■■■■■■■■■■■■■■■■□□□□■■■■■■■■□□□□■■■■■■■	LAM9	42	124326153220224282334132	193
UT469	2006	■■■■■■■■■■■■■■■■■■■■■■■■■□□□□□□■□□□□■■■□■■■	H1	62	225325143323233474234424	222
UT509	2006	■■■■■■■■■■■■■■■■■■■■□□□□■■■■■■■■□□□□■■■■■■■	LAM9	42	124326153220224162434132	229
UT62	2005	■■■■■■■■■■■■■■■■■■■■□□□□■■■■■■■■□□□□■■■■■■■	LAM9	42	124326153220224262334132	187
UT63	2005	■■■■■■■■■■■■■■■■■■■■□□□□■■■■■■■■□□□□■■■■■■■	LAM9	42	124326153220224162334122	186
UT70	2005	■■■■■■■■■■■■■■■■■■■■■■■■■□□□□□□■□□□□■■■□■■■	H1	62	225325153323333574234423	183
