# Supplementary material for: Pan-genome association study of Mycobacterium tuberculosis lineage-4 revealed specific genes related to the high and low prevalence of the disease in patients from the North-Eastern area of Medellín, Colombia
Source: Front Microbiol. 2023 Jan 4;13:1076797. doi: 10.3389/fmicb.2022.1076797 (PMC9846648; doi:10.3389/fmicb.2022.1076797)
Supplement: Supplementary file 11 [file Data_Sheet_2.PDF]

Supplementary Table 2. Genotyping of Low prevalence Mtb Clinical isolates in the northeastern area of Medellín. The spoligotyping shows patterns of presence (black square) or absence (white square) of some of the 43 spacers of the DR locus, as well as the sub-lineage and the number of Spoligo-International-Type (SIT). Each position of the 24-loci MIRU-VNTR represents the number Interspersed Repetitive Units-Variable-Number of Tandem Repeats of a particular locus, useful for discrimination between isolates and the MIRU code to know its geographical location.

Isolate	Year	Spoligotype pattern	Sub-lineage	SIT	24 MIRU-VNTR loci	24-MIT
UT04	2005	■■■■■□■■■■■■■■■■■■■■■■■■■■■■■■□■□□□□■■■■■■■	H3	207	225323153323323573224433	181
UT105	2005	■■■■■■■■■■■■■■■■■■■■■■■□■□□□□□□■□□□□■■■■■■■	H1	45	225325154323323573224133	o
UT125	2005	■■■■■■■■■■■■■■■■■■■■■■■■■■■■■■□■□□□□■■■■■■■	H3	50	225325153323323473124423	o
UT142	2005	■■■■■□■■■■■■■■■■■■■■■■■■■■■■■■□■□□□□■■■■■■■	H3	207	225323153323323573234433	o
UT173	2005	■■■■■■■■■□■■■■□■■■■■□□□□■■■■■■■■□□□□■■□■■■■	LAM9	3040	124326153220224262434132	o
UT222	2005	■■■■■■■■■■■■■■■□■■■■□□□□■■■■■■■■□□□□■■■■■■■	LAM9	1832	224214143321214352234122	o
UT277	2006	■■■□□□□□□□□□■■■■■■■■■■■■■□□□□□□■□□□□■■■□■■■	H1	3022	226325153323333572234423	196
UT303	2006	■■■■■■■■■■■■■■■■■■■■■■■■■■■■■■□■□□□□■■■■■■■	H3	50	225325153223323473224433	221
UT311	2006	■■■■□□□■■■■■■■■■■■■■■■■■■□□□□□□■□□□□■■■■■■■	H1	3043	225325153323323563234433	o
UT323	2006	■■■■■■■■■■■■■■■■■■■■■■■■■■■■■■□■□□□□■■■■■■■	H3	50	225325153223323473224423	o
UT325	2006	■□□■■■■■■■■■■■■■■■■■□□□□■■■■■■■■□□□□■■■■■■■	LAM1	753	224226153221124342234122	o
UT354	2006	■■■■■■■■■■■■■■■■■■■■■■■■■■■■■■□■□□□□■■■■■■■	H3	50	124325153323323573224433	o
UT385	2006	■■■■■■■■■■■■■■□□■■■■■■■■■■■■■■□■□□□□■■■■■■■	H3	1533	225325153323223373324433	o
UT401	2006	■■□■■■■■■■■■■■■■■■■■□□□□■■■■■■■■□□□□■■■□□□□	LAM	3041	224226164221124342234122	219
UT413	2006	■■■■■■■■■■■■■■■■■■■■■■■□■□□□□□□■□□□□■■■■■■■	H1	45	226325153323323572224433	228
UT414	2006	■■■■■■■■■■■■■■■■■■■■■■■□■□□□□□□■□□□□■■■■■■■	H1	45	226325153323323572224433	228
UT419	2006	■■■■■□■■■■■■■■■■■■■■■■■■■■■■■■□■□□□□■■■■■■■	H3	207	225323153323323572224433	0
UT487	2006	■■■■■■■■■■■■■■■■■■■■■■■■■■■■■■□■□□□□■■■■■■■	H3	50	225325153323323573324433	206
UT53	2005	■■■■■■■■■■■■■■■■■■■■■■■□■□□□□□□■□□□□■■■■■■■	H1	45	225325154323323573224433	194
UT86	2005	■■■■■■■■■■■■■■■■■■■■■■■■■■■■■■□■□□□□■■■■■■■	H3	50	225325153323323573224433	184
UT91	2005	□□□□□□■■□□□■■■■■■■■■■□□□□■■■■■■■■□□□□■■■■■■■	LAM3	3311	224225183324222472434211	o
